# Modification of temperature-related human mortality by area-level socioeconomic and demographic characteristics in Latin American cities

**DOI:** 10.1016/j.socscimed.2022.115526

**Published:** 2023-01

**Authors:** Maryia Bakhtsiyarava, Leah H. Schinasi, Brisa N. Sánchez, Iryna Dronova, Josiah L. Kephart, Yang Ju, Nelson Gouveia, Waleska Teixeira Caiaffa, Marie S. O'Neill, Goro Yamada, Sarav Arunachalam, Ana V. Diez-Roux, Daniel A. Rodríguez

**Affiliations:** a Institute of Transportation Studies, University of California, Berkeley, CA, USA; b Urban Health Collaborative, Drexel Dornsife School of Public Health, Philadelphia, USA; c Department of Environmental and Occupational Health, Drexel Dornsife School of Public Health, Philadelphia, USA; d Department of Epidemiology and Biostatistics, Drexel Dornsife School of Public Health, Philadelphia, USA; e Department of Environmental Science, Policy & Management, University of California, Berkeley, USA; f Department of Landscape Architecture & Environmental Planning, University of California, Berkeley, USA; g School of Architecture and Urban Planning, Nanjing University, Nanjing, China; h Department of Preventive Medicine, University of Sao Paulo Medical School, Sao Paulo, Brazil; i Observatório de Saúde Urbana de Belo Horizonte, Universidade Federal de Minas Gerais, Belo Horizonte, Brazil; j Department of Environmental Health Sciences, University of Michigan School of Public Health, Ann Arbor, USA; k Institute for the Environment, University of North Carolina at Chapel Hill, Chapel Hill, USA; l Department of City and Regional Planning and Institute Transportation Studies, University of California, Berkeley, USA

**Keywords:** Temperature-related mortality, Urban health, Latin America, Climate change

## Abstract

**Background:**

In Latin America, where climate change and rapid urbanization converge, non-optimal ambient temperatures contribute to excess mortality. However, little is known about area-level characteristics that confer vulnerability to temperature-related mortality.

**Objectives:**

Explore city-level socioeconomic and demographic characteristics associated with temperature-related mortality in Latin American cities.

**Methods:**

The dependent variables quantify city-specific associations between temperature and mortality: heat- and cold-related excess death fractions (EDF, or percentages of total deaths attributed to cold/hot temperatures), and the relative mortality risk (RR) associated with 1 °C difference in temperature in 325 cities during 2002–2015. Random effects meta-regressions were used to investigate whether EDFs and RRs associated with heat and cold varied by city-level characteristics, including population size, population density, built-up area, age-standardized mortality rate, poverty, living conditions, educational attainment, income inequality, and residential segregation by education level.

**Results:**

We find limited effect modification of cold-related mortality by city-level demographic and socioeconomic characteristics and several unexpected associations for heat-related mortality. For example, cities in the highest compared to the lowest tertile of income inequality have all-age cold-related excess mortality that is, on average, 3.45 percentage points higher (95% CI: 0.33, 6.56). Higher poverty and higher segregation were also associated with higher cold EDF among those 65 and older. Large, densely populated cities, and cities with high levels of poverty and income inequality experience smaller heat EDFs compared to smaller and less densely populated cities, and cities with little poverty and income inequality.

**Discussion:**

Evidence of effect modification of cold-related mortality in Latin American cities was limited, and unexpected patterns of modification of heat-related mortality were observed. Socioeconomic deprivation may impact cold-related mortality, particularly among the elderly. The findings of higher levels of poverty and income inequality associated with lower heat-related mortality deserve further investigation given the increasing importance of urban adaptation to climate change.

## Highlights

• Limited evidence of effect modification of cold-related mortality in Latin American cities.
• High income inequality is associated with greater excess mortality from cold for all ages.
• Segregation and poverty are associated with higher excess mortality due to cold among the elderly.
• Results for heat-related mortality are opposite of expectations (e.g., higher poverty – less heat-related mortality).
• The results for heat-related mortality require further investigation.

## 1 Introduction

Associations of extreme hot and cold ambient temperatures with human mortality have been widely documented in global and regional studies (Zhao et al., 2021; Gasparrini et al., 2015; Tuholske et al., 2021; Green et al., 2019; Campbell et al., 2018; Hajat and Kosatky, 2010; Kephart et al., 2022). An estimated 5 million deaths per year globally are associated with non-optimal temperatures, accounting for 9% of all deaths (Zhao et al., 2021). The temperature-mortality burden varies spatially (Tuholske et al., 2021), with urban areas being particularly vulnerable to extreme heat because of how growing urban populations and the urban heat island effect interact with advancing climate change (Grimm et al., 2008).

An urgent area of research and a public health opportunity is identifying population characteristics that confer vulnerability to (or protection from) heat or cold-related mortality. Multiple cities in Europe and beyond have adopted heat action plans in the aftermath of the 2003 heatwave in Europe that led to an estimated 70,000 excess deaths (Robine et al., 2008). In addition to coordinating response efforts in case of an emergency, these plans help identify vulnerable populations and provide them with necessary care in the event of a heat wave (Robine et al., 2008). The reduction of cold-related mortality also presents a public health opportunity because excess deaths associated with cold outnumber those associated with hot temperatures and make up the majority of temperature-related mortality worldwide (Zhao et al., 2021). Efforts at reducing cold-related mortality have targeted energy insecurity and subpar home insulation (Hajat et al., 2007).

Vulnerability to non-optimal temperatures is a function of exposure, physiological sensitivity, and adaptive capacity (Adger, 2006). Socioeconomic and demographic characteristics relate to each of these pillars of vulnerability. They can modify an individual's vulnerability to heat or cold by influencing the intensity of their exposure to extreme temperatures. For example, persons whose occupations involve working outside may be exposed to high temperatures at disproportionately higher rates compared to those who work inside (Ingole et al., 2017). Ethnic and racial minorities, as well as the poor, tend to live in hotter neighborhoods than non-minorities and the more affluent (Hsu et al., 2021; Jesdale et al., 2013). Furthermore, an individual's socioeconomic position is correlated with the presence of underlying medical conditions (Mielck et al., 2014) and thus shapes biological susceptibility to extreme temperatures (Balbus and Malina, 2009). Socioeconomic characteristics also impact one's adaptive capacity by determining one's ability to achieve thermal comfort when temperatures are extremely hot or cold (e.g., having access to air conditioning or space heaters or weatherized homes) (O'Neill et al., 2005). At the area-level, densely populated and segregated areas may not have adequately equipped and staffed medical facilities to treat temperature-related health episodes, resulting in worse mortality outcomes. Finally, elevated temperatures in densely built-up areas caused by the urban heat island effect may also exacerbate vulnerability to heat (Heaviside et al., 2017).

A number of individual factors have been shown to modify vulnerability to non-optimal temperatures including age (Hajat and Kosatky, 2010; Basu and Ostro, 2008; Gouveia et al., 2003; Medina-Ramón et al., 2006), sex (Ingole et al., 2017; Huang et al., 2015), low socioeconomic position (Huang et al., 2015; Benmarhnia et al., 2015), and pre-existing health conditions (Medina-Ramón et al., 2006; Huang et al., 2015; Benmarhnia et al., 2015; Zanobetti et al., 2013). However, a recent systematic review (Son et al., 2019) reported that the evidence of effect modification of temperature-related mortality by area-level characteristics is limited. In particular, the authors reviewed 207 studies and concluded that the evidence of effect modification by area-level factors, including socioeconomic conditions, is either limited or suggestive (Son et al., 2019). Furthermore, they found weak evidence that factors such as population density, housing quality and availability of healthcare facilities modify the association between temperature and mortality (Gasparrini et al., 2015). Moreover, most studies on the topic originate in North America and Europe (Medina-Ramón et al., 2006; Murage et al., 2020), followed by a few studies in Asia (Huang et al., 2015; Liu et al., 2020; Ng et al., 2016), and a handful of studies in Latin America (Kephart et al., 2022; Gouveia et al., 2003; Zhao et al., 2019; Bell et al., 2008; Son et al., 2016). Little is known about determinants of hot- or cold-temperature vulnerability within middle- and low-income countries and more broadly within the countries of the global South, which have inherently different temperature regimes and may have different adaptive capacities than high income countries (Green et al., 2019; Scheelbeek et al., 2021). We hypothesize that better socioeconomic conditions – low levels of poverty, total mortality, income inequality, residential segregation and better living conditions, as well low population density and small built-up area – are associated with smaller excess mortality attributed to non-optimal temperatures in Latin American cities.

This study uses daily mortality records from 325 cities in nine Latin American countries, combined with daily temperature data and information on cities’ socioeconomic and demographic characteristics to examine the extent to which associations between non-optimal temperatures and mortality are modified by characteristics such as poverty, living conditions, income inequality, and segregation, among others.

## 2 **Methods**

### 2.1 Study area

**Fig. 1:**
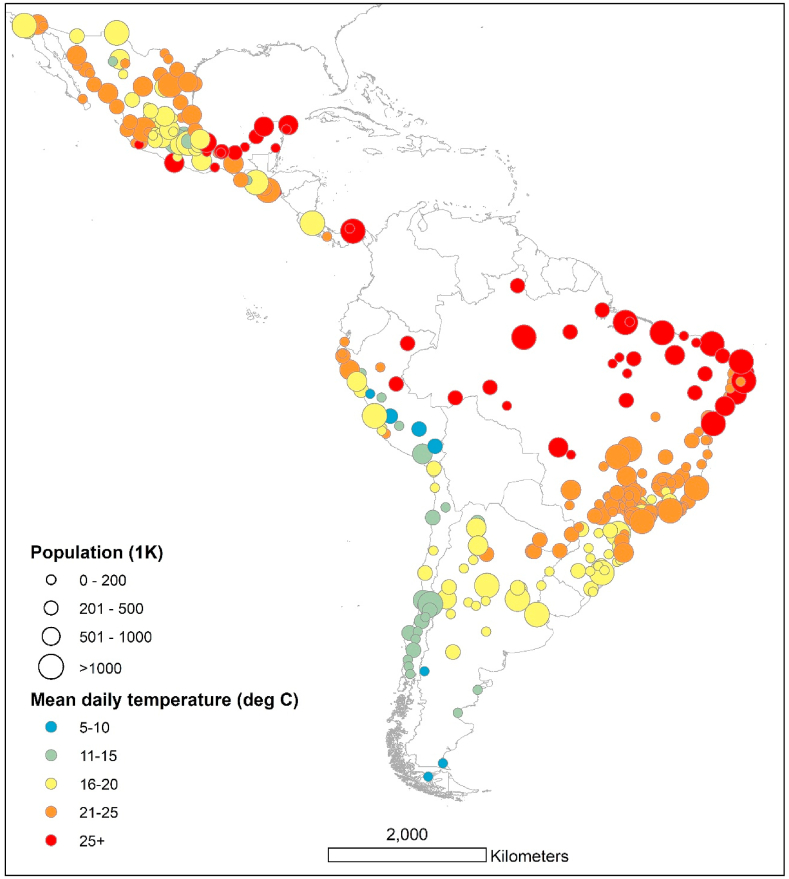
City-specific mean daily temperature and population size.

The study sample consists of cities from the *Salud Urbana en América Latina* (SALURBAL) project. The goal of SALURBAL, an interdisciplinary multinational collaboration, is to investigate the social and environmental determinants of health in Latin American cities (Quistberg et al., 2019). The SALURBAL project includes a total of 371 cities that represent urban agglomerations covering the apparent urban extent or built up area with at least 100,000 residents (Quistberg et al., 2019). This study is based on 325 cities in Argentina, Brazil, Chile, Costa Rica, El Salvador, Guatemala, Mexico, Panama, and Peru. Due to unavailable daily mortality data from Colombia and Nicaragua, cities in these two countries were excluded from the analysis. Fig. 1 depicts the location of the cities used in this study.

### 2.2 Data sources

#### 2.2.1 Mortality data

Individual mortality records were obtained from the vital registration systems in each country for the 2002–2015 period and included date of death, municipality of residence, age at death, and cause of death classified according to the World Health Organization Global Health Estimate (GHE) classifications (World Health Organization, 2021). We conducted analysis for all-cause (GHE tiers I., II., III.) and cardiovascular (II.G.) mortality, as non-optimal temperatures have been associated with cardiovascular deaths (Burkart et al., 2021). Analyses considered deaths at any age, as well as stratification by age at death (<65 and 65+ years).

#### 2.2.2 City characteristics

Guided by the theoretical frameworks on vulnerability to temperature-related mortality (Gronlund, 2014), we extracted and/or calculated (Quistberg et al., 2019) the following city-level characteristics from the countries’ census bureaus, national statistical offices, or analogous organizations: population, population density, age-standardized mortality rate per 100,000 residents, percentage of built up area, living conditions score, secondary education, poverty, Gini index of income inequality, and a measure of residential segregation by education based on isolation index. Age-standardized mortality rate was used as a proxy of the underlying level of population health; lower population health is considered a vulnerability factor for temperature-related mortality (Son et al., 2019). Measures such as living conditions score, poverty, income inequality, and segregation can be thought of as describing socioeconomic deprivation (Bilal et al., 2021). Our measure of residential segregation was based on the isolation index, which characterizes potential contact between social groups, with higher values indicating low levels of exposure of one population group to a different group within an area (Iceland et al., 2002). In all of the sample countries but Brazil, the isolation index measured the extent to which two population groups by educational attainment – those with incomplete primary education vs. university education – are exposed to each other in a city. For Brazil, the isolation index was based on income. More information on the sources of the city-level variables and their computation is available in Quistberg et al. (2019), section “Socioeconomic and demographic predictors” of the Supplementary Material (page 3), and in Supplementary Material Table S1. Table 1 contains descriptions of the city-level characteristics used in the study. The year of measurement for each indicator (varies by country) is presented in Supplementary Material Table S1.

**Table 1:** Description of the socioeconomic and demographic effect modifiers of temperature-related mortality.

Variable name	**Description**^a^	N (cities)
Population	The number of residents in a city.	325
Population density	The number of city residents per km^2^ of city built-up area.	325
Built-up area	Percentage of city area (within the city administrative boundary) classified as built-up by the Global Urban Footprint project.	325
Age-standardized mortality rate	Age-specific all-cause mortality rate weighted by the fraction of population of each age group in the total city population (per 100,000 city residents).	325
Living conditions score	A composite score based on the census measures of:	325
	• Proportion of households with piped water access inside the dwelling;	
	• Proportion of households with more than 3 people per room (reverse coded);	
	• Proportion of the population aged 15 to 17 attending school.	
	The score (Ortigoza et al., 2021) is computed as a sum of z-scores of the constituent variables. Higher values indicate better living conditions.	
Secondary education	Proportion of the population aged 25+ who completed secondary education or above.	325
Poverty	The proportion of the population in the city living in households with household income below the national income poverty line.	319
Gini index	Income-based Gini index of income inequality. Measures the deviation of a country's income distribution from a perfectly equal distribution. The Gini index of ‘0’ signifies perfect equality; ‘1’ means maximum inequality.	296
Segregation	Measured by an index of isolation. The isolation index measures the extent to which a social group resides in neighborhoods where they are exposed only to other members of the same social group (Iceland et al., 2002; Massey and Denton, 1988). In all countries except Brazil, the isolation index compared two population groups: those with primary incomplete education and those with university completed education. The isolation index in Brazil is income based. The index ranges from 0 (complete integration) to 1 (complete segregation). Higher values denote higher levels of segregation.	303

a A table detailing the temporal resolution for each indicator (varies by country) is available in Supplementary Material Table S1. More information about the computation of age-standardized mortality rate and segregation are available in section “Socioeconomic and demographic predictors” in Supplementary Material (p.3). Unless noted otherwise, all the variables were obtained from the census bureaus or population surveys of each country and are described in more detail in Quistberg et al. (2019)

#### 2.2.3 Temperature data and climate zones

Temperature data was obtained from the land surface component of the 5th generation of European ReAnalysis (ERA5), also known as ERA5-Land, produced by the European Centre for Medium-Range Weather Forecasts (ECMWF) and publicly available (Muñoz-Sabater et al., 2021). ERA5-Land supplies hourly temperature at 2 m above land surface for the global extent at 9 × 9 km spatial resolution. Missing ERA5-Land pixels (30% of cities contained ≥1 missing pixels) were imputed using a random forest regression model that included resampled ERA5 temperature (31 km resolution), elevation, and aspect, with further modeling of residuals using kriging for spatial interpolation. For each SALURBAL city we computed population-weighted mean daily temperature for every day during the study period. The computation is described in detail in Kephart et al. (2022)

We obtained data on the prevailing climate zone in a city from the Köppen climate classification (Muñoz-Sabater et al., 2021), which groups the Earth's climates according to temperature and rainfall regimes. Our initial sample of 326 cities from Kephart et al. (2022) included the following climate zones: 79 arid cities, 112 temperate cities, 134 tropical cities, and 1 polar city. We excluded the polar city (Puno in Peru) from the present analysis.

## 3 Statistical analysis

We estimated effect modification of temperature-related mortality by city characteristics in three stages using an approach similar to that in Sera et al. (2019). Specifics of the first and second stage analyses, and their results, are available elsewhere (Kephart et al., 2022). Briefly, we estimated associations between daily mean temperature and daily mortality counts for every city using distributed lag nonlinear conditional Poisson models. We estimated the associations for all-cause deaths and for deaths from cardiovascular disease. The models accounted for lags from 0 to 21 days, and controlled for seasonality by including terms for day of week, month and year (Kephart et al., 2022; Gasparrini, 2014). Next, for each city we obtained summaries that quantified the temperature-mortality associations. To do so, the numerically stabilized (to ensure that cities with too few deaths do not contribute noisy estimates) city-specific non-linear curves of the temperature-mortality association were used to identify the observed optimal temperature from a human health perspective (also known as minimum mortality temperature, or MMT), defined as the daily mean temperature in a city corresponding to the temperature at which mortality rates were the lowest. After deriving MMT from the smoothed curves, we used the raw, unsmoothed city-specific temperature mortality curves to estimate the excess death fractions (EDF). EDFs represent the percent of total deaths observed in a city during the study period that occurred on days with mean temperatures above (heat EDFs) or below (cold EDFs) the MMT, and can be attributed to non-optimal temperature. Extreme heat or extreme cold EDFs refer to the fraction of total deaths that occurred on days on which temperatures were ≥95th percentile or ≤5th percentile of the city-specific daily temperature distribution and can be attributed to non-optimal temperature, respectively. We also estimated the relative risk (RR) of mortality associated with a 1 °C change in city mean daily temperature below the 5th or above the 95th percentile of the city-specific temperature distribution. The relative risk approximates the steepness of the non-linear temperature-mortality curves under extremely hot and extremely cold temperatures. While EDFs describe the relative contribution of non-optimal temperatures to mortality in a city, RRs describe the incremental impact of 1 °C degree lower/higher temperature, for extremely cold/hot temperatures, on mortality. The log relative risk of mortality due to ‘extreme cold’ was computed as the difference in the log-risk of mortality at the 1st and 5th percentiles of city-specific distribution of daily temperatures divided by the difference in degrees Celsius between the 1st and 5th percentiles of the temperature distribution. The log RR due to ‘extreme heat’ was computed similarly: difference between the log-risk of mortality at the 99th and 95th percentiles of the city-specific distribution of daily temperatures divided by the difference in degrees Celsius between the 99th percentile and 95th percentile of the temperature distribution. The resulting estimated relative risk of mortality due to extreme cold, for example, can be interpreted as the relative risk of mortality associated with a 1 °C decrease in mean daily temperature below the 5th percentile of the temperature distribution. Additional details of the first and second stage analyses and results are described and reported elsewhere (Kephart et al., 2022).

Finally, the third stage of our analysis estimated effect modification of the temperature-attributed excess death fractions and the relative risk of mortality by city-level characteristics by implementing random effects meta-regressions (Sera et al., 2019; Gasparrini and Armstrong, 2013). We estimated separate models for each socioeconomic and demographic predictor variable and EDFs and log(RR)s for heat/extreme heat or cold/extreme cold as the dependent variables. Because the dependent variable (EDF or log(RR)) represents an association between temperature and mortality, results from the meta-regressions with city-level characteristics can be interpreted as effect modification estimates. Accordingly, we refer to coefficients from the meta regression for EDFs as the difference in EDF associated with a given socioeconomic characteristic, akin to the interpretation of an interaction term. For the relative risk, we refer to the exponentiated coefficients from the meta regression as interaction relative risks, or proportional difference in RR associated with the given characteristic. To accommodate potential non-linearity of the associations between the levels of city-specific socioeconomic and demographic characteristics and temperature-related mortality, we modeled each predictor measure as a three-level categorical variable, with categories based on tertiles (low, medium, high) of the respective observed distributions. All meta-regressions were adjusted for continuous measures of city daily mean temperature, mean temperature range (measured over all observed timepoints), climate zone (tropical, arid, or temperate), and country (Central American countries were as aggregated into one group) based on the *a priori* hypotheses that these variables might confound associations. The modeling was done using packages “dlnm” (Gasparrini, 2011) and “mvmeta” (Gasparrini et al., 2012) in the R environment for statistical computing (version 4.1.1).

### 3.1 Additional analyses

#### 3.1.1 Analysis by age group, cardiovascular mortality, and climate characteristics

We estimated meta-regressions for the six mortality outcomes (EDF due to cold and heat; EDF due to extreme cold and extreme heat; relative risk of mortality due to extreme cold and extreme heat) among those ages 65+ only. In addition, we also estimated excess deaths associated with hot and cold temperatures for deaths from cardiovascular disease (CVD). Finally, in order to analyze whether effect modification by city characteristics varied depending on the cities’ climatic conditions, we stratified the cities by 1) Köppen climate zones (arid, tropical, and temperate); and 2) category of inter-annual temperature variation (annual temperature range: >10 °C and ≤10 °C).

### 3.2 Sensitivity analysis

We undertook several sensitivity analyses to assess the robustness of the results. First, we compared the results from the all-age models to the results with adjustment for the proportion of population 65 and older as the older age group may disproportionally contribute to mortality relative to its population size. Second, we re-estimated the meta-regressions for each socioeconomic and demographic indicator while adjusting for population and population density to assess the robustness of our results to the city size and population distributions in cities. Third, for each effect modifier we re-estimated the meta-regressions including non-linear spline terms for mean daily temperature and temperature range.

## 4 **Results**

### 4.1 Sample characteristics

Table 2 presents summary statistics for the cities included in the analysis. An analogous country-specific table can be found in Supplementary Material Table S1. Our analysis included a total of 15,360,075 deaths in 325 cities in nine Latin American countries representing over 2.9 billion person-years of risk observed in the analysis. The annual mean temperature varied by city (Fig. 1), with the hottest annual temperatures observed in the cities in Brazil and Central America (median city annual temperatures: 22.2 °C and 23.8 °C, respectively), and the lowest annual temperatures observed in Chilean cities (13.7 °C).

**Table 2:** Descriptive statistics for temperature, mortality, and socioeconomic and demographic variables for the Latin American cities included in the analysis.^a^.

Variable	N cities^b^	Mean	SD	Min	Median	Max
Daily temperature (°C)	325	20.45	4.38	4.93	20.95	27.82
Annual temperature range (°C)	325	14.98	6.73	3.63	14.26	34.25
Minimum Mortality Temperature (°C)	325	23.15	3.22	10.12	23.78	33.47
Total deaths	325	47,262	137,317	2518	16,926	1,594,830
*Socioeconomic and demographic effect modifiers*						
Population (1 K)	325	778	2060	104	271	20,888
Population density per km^2^	325	6,884	2,783	2,446	6,080	23,200
Population >65 years (%)	325	6.70	1.83	1.44	6.59	12.84
Built-up area (%)	325	59.09	6.85	18.72	59.87	71.83
Age-standardized mortality rate per 100,000 residents	325	551.89	119.54	132.94	543.12	942.37
Living conditions score:	325	0.01	1.76	−5.87	0.33	2.31
Households with piped water inside the dwelling (%)	325	87.47	13.14	33.41	92.61	99.71
Overcrowding: households with more than 3 people per room (%)	325	5.96	4.78	0.32	4.18	26.24
15–17 yo attending school (%)	325	79.38	8.17	45.75	82.23	91.96
Secondary education (%)	325	38.70	9.40	13.04	38.39	72.99
Poverty (%)	319	30.62	15.80	4.40	27.80	71.10
Gini index of income inequality	296	0.50	0.08	0.29	0.51	0.68
Segregation (based on isolation index)	303	0.29	0.16	0.09	0.26	0.70

a Supplementary Material Table S1 contains summary statistics by country, including the years of data for every country.

b The number of cities varied by variable because of data availability.

We observed high variability in socioeconomic indicators between the cities in general, as well as between cities within countries. The highest levels of city income inequality were observed in Brazil (median Gini index = 0.55), while in the rest of the countries it hovered around 0.4. According to the living conditions score (a measure describing piped water access in a dwelling, overcrowding, and the proportion of 15–17-year-olds attending school), cities with the most suboptimal living conditions in our sample are located in Central America and Peru. Indeed, the median proportion of households with piped water access inside their dwellings in Peruvian cities is 70%; the proportion of households with piped water access is slightly higher in Central American cities – 76%, compared with 97% in Brazil. Peruvian and Central American cities also had the highest levels of overcrowding, with the median city proportion of households with more than three people per room at about 10%.

#### 4.1.1 Correlations between demographic and socioeconomic indicators

**Fig. 2:**
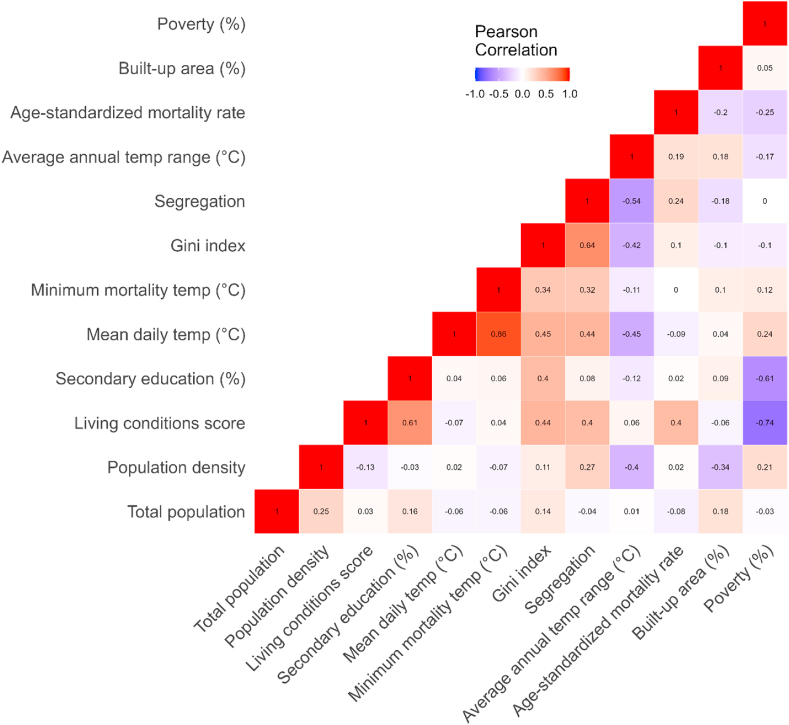
Correlations between pairs of the temperature, socioeconomic, and demographic indicators used in the study.

The demographic and socioeconomic indicators used in the study describe different aspects of cities’ socioeconomic environments, but they exhibit several correlation patterns (Fig. 2). The highest levels of correlation are observed between segregation (isolation index) and the Gini index, as well as between the living conditions score and the Gini index. Poverty prevalence is not strongly correlated with the Gini and segregation measures, meaning that the most unequal and segregated cities are not necessarily the poorest. On the other hand, living conditions are associated with both segregation and the Gini index. The strongest negative correlations are observed between poverty and the living conditions score, which is expected. We observe moderate correlations between population density, segregation, and the Gini index. As Fig. 2 shows, the main exposure variable – mean daily temperature – is strongly and positively correlated with income inequality and isolation index and, expectedly, with the minimum mortality temperature.

### 4.2 Excess mortality associated with temperature

According to a meta-regression of city-specific temperature-mortality associations, 5.75% (95% CI: 5.31, 6.07) of deaths at all ages and from all causes are associated with non-optimal temperatures during the 2002–2015 study period (Kephart et al., 2022). Out of 5.75%, 0.67% (95% CI: 0.58, 0.74) deaths were associated with heat (the cumulative effect of all temperatures above MMT), whereas cold (cumulative effect of temperatures below MMT) accounted for 5.09% (95% CI: 4.64, 5.47) of the deaths (Kephart et al., 2022). The proportion of the observed variance in between-city EDFs that reflects variance in true effect sizes as opposed to error is greater for cold EDFs (I^2^ = 93%) than for heat EDFs (I^2^ = 78). The inclusion of country group reduced I^2^ to 92% for cold EDFs and 76% for heat EDFs. The addition of climate zone, temperature range, and mean daily temperature further reduced I^2^ to 91% for cold EDFs and 72% for heat EDFs.

### 4.3 Effect modification of temperature-related mortality by city-level socioeconomic characteristics

**Fig. 3:**
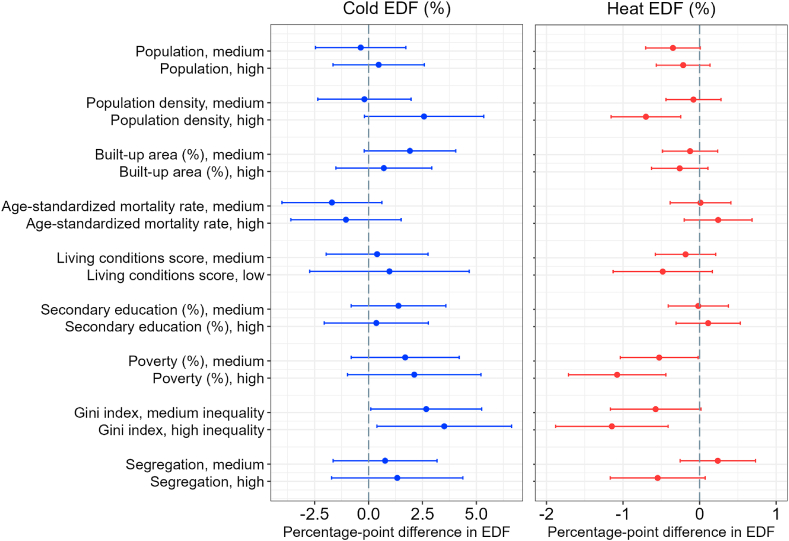
Difference in excess death fractions (EDF) of all-cause mortality associated with cold and hot temperatures by levels of the socioeconomic and demographic characteristics of Latin American cities. Cold temperatures are defined as those below the minimum mortality temperature. Hot temperatures are defined as those above the minimum mortality temperature. Point estimates and 95% confidence intervals are obtained from the random effects meta-regressions that include a socioeconomic indicator, city-level mean daily temperature, mean annual temperature range, climate zone, and country. Separate meta-regressions were fitted for each indicator. The socioeconomic characteristics were categorized as low, medium, and high according to the tertiles of their distribution. The reference category for each effect modifier are cities with desirable levels of the indicator (e.g., low poverty, high living conditions, etc.). In the case of population, population density, and % urban area, the reference are cities with low (bottom tertile) values of these characteristics. Refer to Table 1 for variables' definition. Supplementary Material Table S2 contains the estimates and confidence intervals shown in the figure. The analysis is based on 325 cities for all variables except poverty (n = 319 cities), Gini index (n = 296), and segregation (n = 303).

In this section, we describe associations between city-level socioeconomic and demographic characteristics and: (1) excess death fractions (EDF) related to cold and heat; (2) relative risk (RR) of mortality due to extreme cold and extreme heat. The estimates in Fig. 3 can be interpreted as a percentage-point difference in the EDF associated with differences in city characteristics. The reference category for each indicator are cities with desirable levels of the indicator (e.g., low poverty, high living conditions score, etc.). In the case of population, population density, and % built-up area, the reference are cities with low (bottom tertile) absolute values of these characteristics. All of the results below are adjusted by country, climate zone, mean daily temperature, and average annual temperature range.

#### 4.3.1 Cold-related mortality

The left panel of Fig. 3 (quantitative estimates and confidence intervals are in Supplementary Material Table S2) shows little evidence of effect modification of cold-related mortality as most of the estimates are not statistically significant. The only statistically significant estimate observed is for the Gini index – in cities with high Gini (high income inequality) excess mortality attributable to cold is on average 3.45 percentage-points higher (95% CI: 0.33, 6.56), compared to cities with the low Gini. Estimates for other measures of socioeconomic deprivation such as poverty, living conditions, and segregation are positive – indicating higher cold-related mortality in cities with undesirable levels of these indicators – but confidence intervals were wide and included the null value.

Results for the excess mortality associated with extreme cold (Fig. S1 and Table S3 in the Supplementary Material) showed that cities with the highest Gini index have on average 0.42 percentage point higher EDF from extreme cold (95% CI: 0.05, 0.78) relative to cities with the lowest Gini. All the other associations were not statistically significant.

**Fig. 4:**
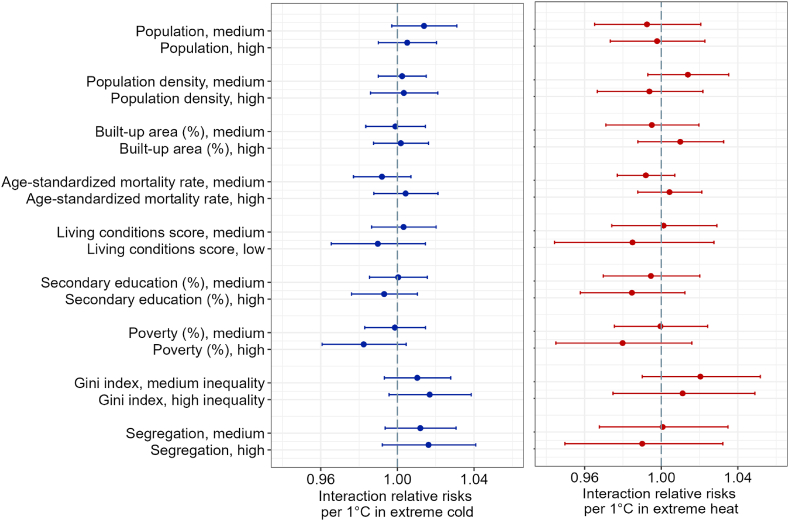
Interaction relative risks (IRRs) of all-cause mortality per 1 °C more extreme cold and extreme hot temperatures by levels of the socioeconomic characteristics of Latin American cities. The IRRs represent proportional difference in RR per 1 °C associated with the given characteristic. RR for extreme cold was computed by dividing the difference in log-relative risk of mortality between temperatures at the 1st and 5th percentile of the city-specific daily mean temperature distribution by the difference in degrees Celsius between the 1st percentile and 5th percentile of the temperature distribution, and exponentiating the quotient. RR for heat was analogously obtained as the difference between the log-relative risk of mortality at the 99th and 95th percentile of the city-specific observed distribution of daily temperatures divided by the difference in degrees Celsius between the 99th percentile and 95th percentile of the temperature distribution, and exponentiating the quotient. For extreme cold, the IRR results can be interpreted as a difference in the relative risk of mortality associated with a 1 °C decrease in mean daily temperature below the 5th percentile of the temperature distribution. For extreme heat, the IRR results present an estimated change in the relative risk of mortality associated with a 1 °C increase in daily mean temperature above the 95th percentile of the temperature distribution. Point estimates and 95% confidence intervals are obtained from the random effects meta-regressions that include a socioeconomic indicator, mean daily temperature, mean annual temperature range, climate zone, and country group. Separate meta-regressions were fitted for each socioeconomic indicator. The reference category for each socioeconomic effect modifier are cities with desirable levels of the indicator (e.g., low poverty, high living conditions score, etc.). In the case of population, population density, and % urban area, the reference are cities with low absolute values (bottom tertile) of these characteristics. The analysis is based on 325 cities for all variables except poverty (n = 319 cities), Gini index (n = 296), and segregation (n = 303). Supplementary Material Table S4 contains the estimates and confidence intervals shown in the figure.

Results for the interaction relative risks of mortality attributed to extreme cold are presented in Fig. 4 (left panel; quantitative estimates and confidence intervals are in Supplementary Material Table S4). These results are not compatible with evidence for effect modification because all the confidence intervals contain the null value.

#### 4.3.2 Heat-related mortality

Associations with EDFs from heat were smaller in magnitude as compared to associations with cold EDFs. As a reminder, heat-related mortality contributed much less than cold-related excess mortality to the overall temperature-related mortality in the study period -- 0.67% (95% CI: 0.58, 0.74) of all temperature-related deaths. High population density and higher socioeconomic deprivation as measured by poverty and income inequality were associated with lower excess mortality due to heat (Fig. 3). Cities in the top tertile of population density have, on average, heat EDF 0.70 percentage points lower (95% CI: 1.16, −0.25) than cities in the bottom tertile. Cities with medium and high poverty, and medium and high Gini index have lower heat EDF relative to cities with desirable levels of these indicators. For example, cities in the top tertile of the Gini index have, on average, heat EDF that is 1.16 percentage points lower (95% CI: 1.90; −0.43) than cities with the smallest Gini index (low income inequality). The estimates for poverty are similar in magnitude.

The effect modifications of the EDF attributable to extreme heat (Fig. S1 and Table S3 in the Supplementary Material) by population density and poverty were in the same direction as those for heat EDF. However, the Gini index was not a statistically significant effect modifier of mortality associated with extreme heat, even though it modified mortality associated with heat. In addition, the EDFs attributable to extreme heat were associated with living conditions and age-standardized mortality rate (ASMR). For example, in cities with high ASMR, extreme heat EDF was 0.28 percentage points higher (95% CI: 0.09; 0.48) compared to cities with low ASMR. Cities with undesirable living conditions score report EDF associated with extreme heat that is 0.31 percentage points lower (95% CI: 0.60; −0.03) compared to cities in the top tertile of the living conditions score.

As for the interaction relative risks of mortality attributed to extreme heat (Fig. 4, right panel), our results showed no evidence that city characteristics are associated with statistically significant proportional differences in the relative risk of mortality per 1 °C increase in mean daily temperature above the 95th percentile.

### 4.4 Additional analyses: age groups, CVD mortality, and city climatic conditions

For those aged 65+ most of the estimates for the socioeconomic effect modifiers of temperature-related mortality did not differ substantially from the above-described results for all ages (Figs. S2–S4 in Supplementary Material). However, the cold-related excess mortality fractions associated with poverty and segregation among 65 and older were greater in magnitude than the cold related excess mortality associated with poverty and segregation in the general population. Specifically, excess mortality associated with cold among the elderly is 6.38 percentage points higher (95% CI: 1.18, 11.59) in the poorest cities and 5.75 percentage points higher (95% CI: 1.24, 10.25) in the most segregated cities, relative to less poor and less segregated cities. The corresponding estimated difference in cold EDFs for all-age mortality do not exceed 3 percentage points (Fig. 3). The unexpected association between heat-related mortality and the Gini index observed for all-age heat-mortality persisted for heat-related mortality among those 65+. Namely, heat EDFs among the elderly in cities with the highest Gini index are, on average, 1.07 percentage points lower (95% CI -1.95; −0.20) compared to the cities with a more even income distribution.

The results for cardiovascular mortality (Fig. S5 in Supplementary Material) demonstrate little evidence of effect modification by city-level indicators.

We did not observe substantial differences in the associations between temperature-related mortality (both for excess death fractions and interactions relative risk) and city characteristics among cities in arid, temperate, and tropical climate zones (Figs. S6–S8 in the Supplementary Material), as well as in cities with different levels of mean annual temperature range (results not shown). Sensitivity analyses (Figs. S9–S11 in Supplementary Material) showed the robustness of our results.

## 5 Discussion

### 5.1 Evidence for effect modification

Overall, our results show limited effect modification of cold-related mortality in Latin American cities by city-level demographic and socioeconomic characteristics. The Gini index of income inequality is the only modifier that exhibited statistically significant associations with all-age cold-related mortality: among all-cause all-age mortality, cities with the high Gini index reported higher excess mortality due to cold compared to the reference cities with low values of the Gini index (more equal income distribution). Other indicators including segregation and poverty were also associated with higher excess mortality due to cold especially among the elderly (65+). We find no clear pattern of effect modification for the relative risk of mortality associated with a 1 °C decrease in temperature below the 5th percentile.

We observe more examples of effect modification of excess mortality associated with heat, however, many of those associations are puzzling and opposite of our expectations. For example, cities with high income inequality and high poverty have lower heat EDFs compared to cities with desirable levels of these indicators. While the finding of high age-standardized mortality rate associated with larger excess mortality due to extreme heat (compared to cities with low age-standardized mortality rate) was in line with our *a priori* hypothesis, the findings that cities with high population density, low living conditions scores, and medium and high poverty have smaller excess mortality from extreme heat (compared to cities with low population density and cities with desirable living conditions score and low poverty) were not expected. We find no modification of the relative risk of mortality associated with a 1 °C increase in temperature above the 95th percentile.

Although we hypothesized that desirable socioeconomic characteristics – such as low levels of poverty, income inequality, residential segregation, and high standards of living conditions, as well as small % built-up, low population, and low population density – would be associated with smaller excess mortality and smaller relative risk of mortality due to non-optimal temperatures, our findings show only limited support for these pathways in the case of cold-related mortality and unexpected directions of the associations for heat-related mortality.

### 5.2 Evidence from existing studies

While multiple studies have demonstrated the contribution of higher- and lower-than-optimal temperatures to mortality all over the globe (Zhao et al., 2021; Gasparrini et al., 2015; Kephart et al., 2022), to date few studies have analyzed effect modification of temperature-related mortality, and the analyses of effect modification outside of the Global North are even scarcer (Green et al., 2019). Still, our finding of no large-scale effect modification of temperature-related mortality by city-level socioeconomic characteristics is in line with several other studies based in the Europe (Murage et al., 2020; Åström et al., 2020), Asia (Barnard et al., 2008), Latin America (Gouveia et al., 2003; Bell et al., 2008), and Australia (Yu et al., 2010). A study by Sera et al. (2019) included 37 cities from Brazil, Mexico, Chile and Colombia alongside 303 cities from Southeast Asia and the Global North. The authors also found no effect modification of excess deaths fractions associated with cold temperatures by any city-level characteristics they considered, including unemployment rate, gross domestic product, poverty, Gini index, hospital bed rates, and life expectancy, among others. However, the authors found that higher income inequality (Gini index), population, and population density were associated with larger fractions of deaths attributed to heat, whereas we find the opposite. It is worth mentioning that Sera et al. modeled the above-mentioned city characteristics as continuous variables whereas we modeled them as categorical variables with three levels to detect non-linear associations; the definitions of cities in our studies may also differ. Among the studies based in Latin America, a study from Sao Paulo by Gouveia et al. (2003) found no effect modification of the relative risk of mortality due to cold and heat by a composite index based on income, educational attainment, and living conditions. An analysis of vulnerability to heat-related mortality in Sao Paulo, Santiago, and Mexico City found that the vulnerability by education and sex varied widely between cities (Bell et al., 2008). Another study from a warm city – Hong Kong – showed a 0.65% increase in the relative risk of mortality may be associated with a 3 °C (95% CI: 0.998–1.909) increase in average daily temperature above 28.2 °C for areas of Hong Kong with low socioeconomic status (Liu et al., 2020).

Some of our results are also in contrast to several studies from China (Huang et al., 2015), Japan (Ng et al., 2016), and France (Benmarhnia et al., 2014), among others, that found higher cold-related (Huang et al., 2015) and heat-related (Ng et al., 2016; Benmarhnia et al., 2014) mortality in socioeconomically deprived areas. Results from a study based in Mexico revealed lower vulnerability to cold-related mortality among individuals in the top quartiles of personal income distribution, although no effect modification for heat was found (Cohen and Dechezleprêtre).

Our study is among the first to explore city socioeconomic characteristics as effect modifiers of temperature-related mortality in Latin America, so, barring a few exceptions we cannot compare the observed results with other studies in the same region. However, the use of community- or area-wide effect modifiers, which ignore within-city and between-person or -neighborhood heterogeneity in socioeconomic wellbeing, potentially contributes to the findings of no effect modification (Gouveia et al., 2003; Åström et al., 2020; Barnard et al., 2008). Future work should examine temperature-related mortality in relation to within-city heterogeneity in socioeconomic indicators.

### 5.3 Explaining counter-intuitive results for heat-related mortality

The finding of lower heat EDF in cities with high Gini index and high poverty is in contrast to our expectation and requires replication and confirmation. Cities in the highest 30% of income inequality as measured by Gini index are almost exclusively concentrated in Brazil (96 cities in Brazil and 6 cities in Mexico comprise the top tertile of the high-Gini cities). Similarly, 30 cities in Mexico and 71 cities in Brazil account for 98% of the poorest cities in the sample. These highly unequal and poor cities also tend to be the hottest cities in our entire sample. In fact, the correlations between mean daily temperature and city characteristics are the highest for Gini (r = 0.45) and poverty (r = 0.24), of all the city characteristics. Our controls for mean daily temperature, temperature range, and climate zone account for the confounding effect of temperature. It is possible, however, that hotter cities (which also tend to be the poorest and most unequal cities in our sample) are more adapted to high temperatures and that adjusting for daily temperature, range and climate zone does not directly capture this adaptation. To explore this, we estimated associations between MMT and city-level predictors (Supplementary Material Table S10). These results show that higher Gini index (higher income inequality) is associated with higher MMT: in cities with high Gini index MMT was on average 0.7 °C higher than in low-Gini index (low inequality) cities, adjusted for country group, mean daily temperature, annual average temperature range, and climate zone. (The only other statistically significant association was observed for % built-up; cities with moderate levels of built-up have lower MMTs compared to cities with low % built-up area.) This preliminary finding suggests that higher-inequality cities may be more adapted to higher temperatures. However, this exploratory analysis does not help explain the puzzling associations between poverty and heat-related mortality. Future research should confirm this and investigate the role of adaptation in temperature-mortality associations in a more comprehensive manner.

Another potential explanation may lie in the distribution of greenspace. A recent study showed that in the same Latin American cities as in our study, city socioeconomic status (as measured by the Gross Domestic Product per capita and a composite index of social environment) (Bilal et al., 2021) is negatively associated with city-level greenness (cities with lower socioeconomic status tend to have more abundant greenspace compared to cities with higher socioeconomic status) (Ju et al., 2021). The abundance of greenspace in cities that are worse off socioeconomically may reduce vulnerability to heat-related mortality by providing cooling. Introducing percentage of city area covered by greenspace as an additional meta-predictor did not change the effect modification estimates for the Gini and poverty, but investigating greenspace as an effect modifier requires a more complex approach that is outside of the scope of this paper and is currently underway by our team.

Overall, the observed counterintuitive relationships for heat-related mortality may be due to high levels of acclimatization to higher temperatures among the highly unequal and poor cities. Future research should identify measures of and account for the role of population adaptive capacity in vulnerability to non-optimal temperatures.

### 5.4 Implications for public health in the face of climate change

Despite limited evidence of modification of cold-related mortality and unexpected patterns of modification of heat-related mortality by city socioeconomic characteristics, age-standardized mortality rate emerged as an effect modifier of excess mortality due to extreme heat, and Gini index of income inequality emerged as a modifier of cold-related mortality. In this study we used city-specific age-standardized mortality rate as a measure of population health, which may be correlated with higher levels of susceptibility to temperature-related mortality because of underlying chronic conditions. This may suggest that the strategies and interventions that cities are pursuing to reduce mortality at large should also work for reducing mortality associated with extreme heat. Empirical evidence for an increased risk of mortality due to heat among people with pre-existing health conditions such as diabetes, Alzheimer's disease, and dementia has been reported in existing research (Zanobetti et al., 2013; Geirinhas et al., 2020). A study based in Rio de Janeiro, Brazil (Geirinhas et al., 2020), found diabetic illnesses to be the largest contributor to excess temperature mortality. In this case, reducing the prevalence of diabetes could also reduce temperature-related mortality. The authors of a Mexico-based study found that the rollout of Mexico's universal health insurance program called *Seguro Popular* was associated with a 30% decrease in cold-related deaths among the poor, possibly by increasing medical assistance in general as well as expanding insurance coverage to include winter-related illnesses such as pneumonia (Cohen and Dechezleprêtre). Thus, in the face of limited resources, reducing the prevalence of underlying chronic diseases, which would in turn decrease age-standardized mortality rate, will likely be beneficial for reducing temperature-related mortality as well.

Our findings suggest associations between 1) income inequality and higher cold-related mortality for all ages; 2) poverty and segregation and higher cold-related mortality among the elderly. Population's adaptive capacity to withstand the impact of cold and extreme cold may be bolstered by expanding the use of heaters in dwellings and weatherizing homes.

### 5.5 Limitations

First, between-individual or neighborhood variability in socioeconomic characteristics is likely much higher than the between-city variability exploited in the study. Therefore, an analysis using individual-level mortality outcomes and individual-level socioeconomic characteristics would provide a more robust assessment of effect modification. Relatedly, within-city variability in socioeconomic characteristics is also likely higher than the between-city variability, so an alternative analysis could utilize socioeconomic and demographic data from a level smaller than city – such as neighborhood level, for example. It may be that the temperature-mortality relationship is modified by socioeconomic environment at the neighborhood level, but less so at the highly heterogeneous city level. Second, no variables describing healthcare provision and accessibility were available for this analysis, whereas the number of hospital beds (Sera et al., 2019) and access to health insurance (Cohen and Dechezleprêtre) may be associated with vulnerability to mortality from extreme temperature exposures. Third, our study did not adjust for nor considered atmospheric humidity as a possible effect modifier. Fourth, while the mortality data covered the period from 2002 to 2015, the effect modifiers used in the analysis were cross-sectional. Fifth, we utilized city-level measures of temperature, which is an approximation of an individual exposure to temperature. Sixth, a recent study (Avashia et al., 2021) found that land surface temperature may produce statistically different estimates of mortality risk compared to air temperature, which is used in most temperature-mortality studies. Land surface temperature may represent a better exposure metric for evaluating mortality risk attributed to temperature as it more accurately reflects localized thermal environment, especially in an urban context where dense build-up and high concentrations of impervious surfaces contribute to the heat island effect (Avashia et al., 2021). Seventh, because we estimated separate models for each city-level characteristic, our statistically significant results should be interpreted with caution due to the issue of multiple comparisons. Finally, it is worth noting that limited variability in between-city heat EDFs may be a limiting factor in detecting conclusive patterns of effect modification for heat-related mortality, since the between-city variability in cold EDF is higher than the variability in heat.

### 5.6 Strengths

This work explored effect modification of mortality associated with both hot and cold non-optimal temperatures, using excessive death fractions as well as RR per degree difference at temperatures above the 95th percentile and below the 5th percentile. We used population-weighted air temperature, which enabled a more accurate measurement of city-level exposure compared to temperature measures that do not account for population distribution. Our study based on individual mortality records from a large and diverse sample is among the first studies of temperature-related mortality in Latin America, an extremely understudied region. While many previous studies of temperature-mortality and its effect modification by area-level socioeconomic characteristics considered a limited number of cities in Latin America and the Global South (Zhao et al., 2021; Sera et al., 2019), cities outside of the Global North have represented a small fraction of the total sample. This study provides evidence for effect modification of temperature-related mortality for hundreds of cities in Latin America's temperate and tropical regions, which have distinct temperature and precipitation regimes from those in the Global North.

### 5.7 Conclusion

Overall, we find limited effect modification of cold-related mortality in Latin American cities by city-level demographic and socioeconomic characteristics, and several unexpected patterns of effect modification of heat-related mortality. Cold-related mortality in Latin American cities may be impacted by income inequality, particularly among the elderly. The findings of higher levels of poverty and income inequality associated with lower heat-related mortality deserve further investigation. Future studies should consider measures of adaptive capacity when investigating vulnerability to non-optimal temperatures.

## Author contribution

All authors conceptualized the analysis. MB and BNS completed formal analysis. MB created the original draft of the manuscript. ADR and DAR are senior investigators and provided supervision. All authors contributed to writing and manuscript editing.

## Code availability

Requests for the code used in this study should be made to the corresponding author.

## Declaration of competing interest

The authors declare no competing interests.

## Data Availability

Data will be made available on request.
