# Supplementary material for: Modification of temperature-related human mortality by area-level socioeconomic and demographic characteristics in Latin American cities
Source: Soc Sci Med. Author manuscript; Available in PMC 2023 Feb 15. (PMC9870751; doi:10.1016/j.socscimed.2022.115526)
Supplement: Supplementary Data [file EMS158118-supplement-Supplementary_Data_.docx]

# **Contents**

# Socioeconomic and demographic predictors.

# Table S1. Summary statistics by country

# Table S2. Figure 2. Differences in excess death fractions (EDF) of all-cause mortality associated with cold and hot temperatures by levels of the socioeconomic and demographic characteristics of Latin American cities.

# Figure S1. Differences in excess death fractions (EDF) of all-cause mortality associated with extremely cold and extremely hot temperatures by levels of the socioeconomic and demographic characteristics of Latin American cities.

# Table S3. Differences in excess death fractions (EDF) of all-cause mortality associated with extremely cold and extremely hot temperatures by levels of the socioeconomic and demographic characteristics of Latin American cities.

# Table S4. Interaction relative risks (IRRs) of mortality attributed to extremely cold and extremely hot temperatures by levels of the socioeconomic and demographic characteristics.

# Figure S2. Differences in excess death fractions (EDF) associated with cold and extremely cold temperatures for population 65 years and older by levels of the socioeconomic and demographic characteristics of Latin American cities.

# Figure S3. Differences in excess death fractions (EDF) associated with hot and extremely hot temperatures for population 65 years and older by levels of the socioeconomic and demographic characteristics of Latin American cities

# Figure S4. Interaction relative risks (IRRs) of mortality among the population 65 years and older attributed to extremely cold and extremely hot temperatures by levels of the socioeconomic characteristics of Latin American cities.

# Figure S5. Differences in excess death fractions (EDF) of cardiovascular mortality associated with cold and hot temperatures by levels of the socioeconomic characteristics of Latin American cities.

# Table S5. Differences in excess death fractions (EDF) due to cold, hot, extremely cold, and extremely hot temperatures among the population ages 65+ by levels of the socioeconomic characteristics of Latin American cities.

# Table S6. Interaction relative risks (IRRs) of all-cause mortality attributed to extremely cold and extremely hot temperatures among the population ages 65+ by levels of the socioeconomic characteristics of Latin American cities.

# Table S7. Differences in excess death fractions (EDF) due to cold and hot temperatures for all ages for cardiovascular deaths by levels of the socioeconomic characteristics of Latin American cities.

# Figures S6-S8. Differences in excess death fractions (EDF) due to cold and hot temperatures stratified by climate zone.

# Figures S9-S10. Differences in excess death fractions (EDF) of all-cause mortality associated with cold, hot, extremely cold and extremely hot temperatures by levels of the socioeconomic and demographic characteristics of Latin American cities, adjusted by the proportion of city population 65 years and older.

# Table S8. Differences in excess death fractions (EDF) of all-cause mortality associated with cold, hot, extremely cold and extremely hot temperatures by levels of the socioeconomic and demographic characteristics of Latin American cities, adjusted by the proportion of city population 65 years and older.

# Figure S11. Interaction relative risks (IRRs) of mortality attributed to extremely cold and extremely hot temperatures by levels of the socioeconomic and demographic characteristics, adjusted by the proportion of city population 65 years and older.

# Table S9. Interaction relative risks (IRRs) of mortality attributed to extremely cold and extremely hot temperatures by levels of the socioeconomic and demographic characteristics, adjusted by the proportion of city population 65 years and older.

# Socioeconomic and demographic predictors.

As mentioned in the main text, the majority of variables were directly obtained from the country-specific census or population surveys. However, the age-standardized mortality rate was computed by the paper’s authors directly, while the measure of segregation was computed by the larger SALURBAL project.

1.a. We computed age-standardized mortality rate (ASMR) for city *i* and year *t* as:

${AMSR}_{it}$= ( $\frac{D_{it 0}}{N_{it 0}}{*P}_{it 0}$) + ( $\frac{D_{it 1-4}}{N_{it 1-4}}$ ${*P}_{it 1-4}$) + … + ($\frac{D_{it 65+}}{N_{it 65+}}{*P}_{it 65+}$) , where

- D_it 0_ is the number of deaths among individuals up to one year of age (infants) in city *i* in year *t*; D_it 1-4_ is the number of deaths for the people ages 1-4, and so forth until D_it 65+_ (65+ age group). The age groups included are: infants up to 1 year old, 1-4, 5-19, 20-34, 35-49, and 65+.
- N_it 0_ is the total number of infants in city *i* in year *t*; N_it 1-4_ is the total number of people ages 1-4, and so forth until N_it 65+_ (65+ age group).
- P_it_ is the proportion of the population belonging to each age group (population weight) in city *i* in year *t*: P_it 0_ is the proportion infants in the total population of city *i* in year *t*; P_it 1-4_ is the proportion of those ages 1-4 in the population of city *i* in year *t*, and so forth until P_65+._

For every city, the final ASMR used in the analysis represented an average of year-specific ASMRs. City-specific years for which the ASMR was computed correspond to the available years of mortality data and are listed in Table S1 below.

The mortality data used to compute ASMR is described in the main text.

1.b. A measure of residential segregation is based on isolation index, computed as^1^

$$Isolation index= \sum_{i=1}^{n} \frac{x_{i}}{X_{t}}* \frac{x_{i}}{t_{i}}$$

Where x_i_ is the number of people in group x in the i^th^ sub-city unit (neighborhood/census tract); *X_T_* is the total number of group *X* in the city; and *t_i_* is total population in the *i*^th^ unit (neighborhood/census tract).

Isolation index measures the extent to which members of one minority group are exposed to the members of their own group, as opposed to members of other groups within a city.

For all countries but Brazil, the isolation indices represented education-based segregation, based on the educational attainment of people 25 years or older (in Mexico, those of 15 years or older). The two groups were:

- Those with primary incomplete education
- Those with completed university education

The income-based segregation was available for Brazil. The isolation index was calculated for the following income groups: ≤2 minimum wages vs. others.

All the data to compute the isolation index were obtained from the country-specific census bureaus.

References

1. Massey, D. S. & Denton, N. A. The dimensions of residential segregation. *Soc. Forces* **67**, 281–315 (1988).

Table S1. Summary statistics by country

| Variable | Country | Mean | Standard deviation | Min | Median | Max | N (cities) | Years |
| --- | --- | --- | --- | --- | --- | --- | --- | --- |
| Total deaths | Argentina | 27,378 | 36,133 | 3,092 | 15,638 | 172,311 | 28 | 2009-2015 |
| Annual temperature range (°C) | Argentina | 25.806 | 2.283 | 20.513 | 25.840 | 29.963 | 28 | 1996-2015 |
| Daily temperature (°C) | Argentina | 16.887 | 3.840 | 6.218 | 17.055 | 22.467 | 28 | 1996-2015 |
| Minimum Mortality Temperature (°C) | Argentina | 23.115 | 2.808 | 16.730 | 23.790 | 27.331 | 28 | 2009-2015 |
| Age-standardized mortality rate per 100K residents | Argentina | 650.243 | 137.881 | 232.288 | 653.555 | 942.369 | 28 | 2009-2015 |
| Gini index | Argentina | 0.396 | 0.032 | 0.332 | 0.395 | 0.453 | 28 | 2017 |
| Segregation | Argentina | 0.176 | 0.036 | 0.113 | 0.177 | 0.249 | 28 | 2010 |
| Population | Argentina | 866,252 | 2,745,406 | 87,887 | 238,115 | 14,768,481 | 28 | 2009-2015 |
| Population >65 (%) | Argentina | 8.934 | 1.818 | 6.070 | 8.232 | 12.844 | 28 | 2009-2015 |
| Population density per km2 | Argentina | 5,492 | 1,316 | 3,275 | 4,999 | 8,247 | 28 | 2009-2015 |
| Poverty (%) | Argentina | 27.304 | 6.940 | 14.100 | 26.700 | 41.800 | 23 | 2016 |
| Living conditions score | Argentina | 0.315 | 1.023 | -1.794 | 0.627 | 1.979 | 28 | 2010 |
| Households with piped water inside the dwelling (%) | Argentina | 91.040 | 5.029 | 77.409 | 91.833 | 97.649 | 28 | 2010 |
| Overcrowding: households with more than 3 people per room (%) | Argentina | 4.209 | 1.342 | 2.062 | 3.821 | 7.116 | 28 | 2010 |
| 15-17 yo attending school (%) | Argentina | 83.540 | 3.999 | 74.009 | 83.990 | 91.957 | 28 | 2010 |
| Secondary education (%) | Argentina | 37.179 | 4.023 | 27.710 | 37.066 | 47.794 | 28 | 2010 |
|  |  |  |  |  |  |  |  |  |
| Total deaths | Brazil | 59,428 | 172,109 | 7,419 | 20,208 | 1,594,830 | 152 | 2002-2015 |
| Annual temperature range (°C) | Brazil | 13.197 | 4.866 | 3.775 | 13.750 | 25.087 | 152 | 1996-2015 |
| Daily temperature (°C) | Brazil | 22.256 | 2.740 | 15.903 | 22.087 | 27.817 | 152 | 1996-2015 |
| Minimum Mortality Temperature (°C) | Brazil | 24.191 | 1.752 | 19.124 | 24.102 | 28.711 | 152 | 2002-2015 |
| Age-standardized mortality rate per 100K residents | Brazil | 593.612 | 103.354 | 298.297 | 589.646 | 850.024 | 152 | 2002-2015 |
| Gini index | Brazil | 0.554 | 0.046 | 0.454 | 0.547 | 0.683 | 152 | 2010 |
| Segregation | Brazil | 0.407 | 0.130 | 0.145 | 0.386 | 0.699 | 152 | 2010 |
| Population | Brazil | 608,878 | 1,809,721 | 51,725 | 181,112 | 18,580,718 | 152 | 2002-2015 |
| Population >65 (%) | Brazil | 6.846 | 1.634 | 2.061 | 6.949 | 10.404 | 152 | 2002-2015 |
| Population density per km2 | Brazil | 6,513 | 2,499 | 3,022 | 5,679 | 20,278 | 152 | 2002-2015 |
| Poverty (%) | Brazil | 25.438 | 13.278 | 5.600 | 21.800 | 63.100 | 152 | 2010 |
| Living conditions score | Brazil | -0.470 | 2.004 | -5.872 | -0.363 | 2.187 | 152 | 2010 |
| Households with piped water inside the dwelling (%) | Brazil | 94.701 | 6.524 | 65.011 | 97.608 | 99.707 | 152 | 2010 |
| Overcrowding: households with more than 3 people per room (%) | Brazil | 2.926 | 2.344 | 0.320 | 2.422 | 12.974 | 152 | 2010 |
| 15-17 yo attending school (%) | Brazil | 84.162 | 3.364 | 72.591 | 84.307 | 90.528 | 152 | 2010 |
| Secondary education (%) | Brazil | 39.905 | 6.262 | 22.217 | 39.732 | 57.465 | 152 | 2010 |
|  |  |  |  |  |  |  |  |  |
| Total deaths | Chile | 36,962 | 82,136 | 7,436 | 13,061 | 387,651 | 21 | 2004-2015 |
| Annual temperature range (°C) | Chile | 16.748 | 4.425 | 8.587 | 16.507 | 23.251 | 21 | 1996-2015 |
| Daily temperature (°C) | Chile | 13.408 | 2.808 | 4.934 | 13.702 | 17.377 | 21 | 1996-2015 |
| Minimum Mortality Temperature (°C) | Chile | 19.503 | 2.647 | 10.770 | 19.561 | 19.503 | 21 | 2004-2015 |
| Age-standardized mortality rate per 100K residents | Chile | 548.984 | 75.905 | 396.347 | 527.263 | 672.502 | 21 | 2004-2015 |
| Gini index | Chile | 0.408 | 0.035 | 0.329 | 0.403 | 0.476 | 21 | 2015 |
| Segregation | Chile | 0.219 | 0.057 | 0.126 | 0.219 | 0.319 | 21 | 2002 |
| Population | Chile | 479,550 | 1,264,955 | 74,413 | 144,983 | 5,964,536 | 21 | 2004-2015 |
| Population >65 (%) | Chile | 8.712 | 1.529 | 5.430 | 8.736 | 11.616 | 21 | 2004-2015 |
| Population density per km2 | Chile | 7,430 | 1,930 | 5,101 | 6,783 | 12,700 | 21 | 2004-2015 |
| Poverty (%) | Chile | 10.867 | 4.544 | 4.400 | 10.800 | 19.600 | 21 | 2015 |
| Living conditions score | Chile | 1.556 | 0.723 | -0.919 | 1.792 | 2.308 | 21 | 2002 |
| Households with piped water inside the dwelling (%) | Chile | 95.196 | 3.354 | 85.164 | 95.709 | 99.231 | 21 | 2002 |
| Overcrowding: households with more than 3 people per room (%) | Chile | 3.807 | 1.147 | 1.666 | 3.537 | 6.922 | 21 | 2002 |
| 15-17 yo attending school (%) | Chile | 83.290 | 2.661 | 75.246 | 83.550 | 87.020 | 21 | 2002 |
| Secondary education (%) | Chile | 41.745 | 4.689 | 31.496 | 42.563 | 49.389 | 21 | 2002 |
|  |  |  |  |  |  |  |  |  |
| Total deaths | Costa Rica | 64,117 | NA | 64,117 | 64,117 | 64,117 | 1 | 2010-2015 |
| Annual temperature range (°C) | Costa Rica | 5.003 | NA | 5.003 | 5.003 | 5.003 | 1 | 1996-2015 |
| Daily temperature (°C) | Costa Rica | 19.199 | NA | 19.199 | 19.199 | 19.199 | 1 | 1996-2015 |
| Minimum Mortality Temperature (°C) | Costa Rica | 19.758 | NA | 19.758 | 19.758 | 19.758 | 1 | 2010-2015 |
| Age-standardized mortality rate per 100K residents | Costa Rica | 443.250 | NA | 443.250 | 443.250 | 443.250 | 1 | 2010-2015 |
| Gini index | Costa Rica | NA | NA | NA | NA | NA | NA | NA |
| Segregation | Costa Rica | 0.160 | NA | 0.160 | 0.160 | 0.160 | 1 | 2011 |
| Population | Costa Rica | 1,926,467 | NA | 1,926,467 | 1,926,467 | 1,926,467 | 1 | 2010-2015 |
| Population >65 (%) | Costa Rica | 7.394 | NA | 7.394 | 7.394 | 7.394 | 1 | 2010-2015 |
| Population density per km2 | Costa Rica | 7,104 | NA | 7,104 | 7,104 | 7,104 | 1 | 2010-2015 |
| Poverty (%) | Costa Rica | NA | NA | NA | NA | NA | NA | NA |
| Living conditions score | Costa Rica | -0.480 | NA | -0.480 | -0.480 | -0.480 | 1 | 2011 |
| Households with piped water inside the dwelling (%) | Costa Rica | 99.371 | NA | 99.371 | 99.371 | 99.371 | 1 | 2011 |
| Overcrowding: households with more than 3 people per room (%) | Costa Rica | 0.552 | NA | 0.552 | 0.552 | 0.552 | 1 | 2011 |
| 15-17 yo attending school (%) | Costa Rica | 80.423 | NA | 80.423 | 80.423 | 80.423 | 1 | 2011 |
| Secondary education (%) | Costa Rica | 42.950 | NA | 42.950 | 42.950 | 42.950 | 1 | 2011 |
|  |  |  |  |  |  |  |  |  |
| Total deaths | El Salvador | 20,606 | 22,421 | 6,601 | 8,751 | 46,465 | 3 | 2009-2014 |
| Annual temperature range (°C) | El Salvador | 7.933 | 1.068 | 6.717 | 8.357 | 8.723 | 3 | 1996-2015 |
| Daily temperature (°C) | El Salvador | 24.739 | 2.142 | 23.209 | 23.821 | 27.188 | 3 | 1996-2015 |
| Minimum Mortality Temperature (°C) | El Salvador | 25.641 | 1.590 | 23.805 | 26.524 | 26.594 | 3 | 2009-2014 |
| Age-standardized mortality rate per 100K residents | El Salvador | 572.534 | 89.176 | 501.599 | 543.360 | 672.644 | 3 | 2009-2014 |
| Gini index | El Salvador | 0.407 | 0.019 | 0.388 | 0.409 | 0.425 | 3 | 2016 |
| Segregation | El Salvador | 0.481 | 0.104 | 0.365 | 0.513 | 0.566 | 3 | 2007 |
| Population | El Salvador | 654,448 | 790,579 | 194,760 | 201,263 | 1,567,322 | 3 | 2009-2014 |
| Population >65 (%) | El Salvador | 7.770 | 0.354 | 7.399 | 7.804 | 8.106 | 3 | 2009-2014 |
| Population density per km2 | El Salvador | 12,004 | 1,759 | 10,153 | 12,205 | 13,653 | 3 | 2009-2014 |
| Poverty (%) | El Salvador | 31.367 | 10.768 | 25.100 | 25.200 | 43.800 | 3 | 2016 |
| Living conditions score | El Salvador | -1.103 | 1.362 | -2.265 | -1.438 | 0.396 | 3 | 2007 |
| Households with piped water inside the dwelling (%) | El Salvador | 74.215 | 9.023 | 66.334 | 72.252 | 84.057 | 3 | 2007 |
| Overcrowding: households with more than 3 people per room (%) | El Salvador | 11.373 | 1.425 | 10.370 | 10.745 | 13.005 | 3 | 2007 |
| 15-17 yo attending school (%) | El Salvador | 70.194 | 6.988 | 63.814 | 69.105 | 77.662 | 3 | 2007 |
| Secondary education (%) | El Salvador | 29.499 | 6.555 | 24.814 | 26.693 | 36.990 | 3 | 2007 |
|  |  |  |  |  |  |  |  |  |
| Annual deaths | Guatemala | 48,475 | 68,035 | 7,720 | 10,688 | 127,016 | 3 | 2009-2015 |
| Annual temperature range (°C) | Guatemala | 6.609 | 1.862 | 5.307 | 5.779 | 8.742 | 3 | 1996-2015 |
| Daily temperature (°C) | Guatemala | 18.804 | 4.651 | 14.275 | 18.569 | 23.569 | 3 | 1996-2015 |
| Minimum Mortality Temperature (°C) | Guatemala | 20.294 | 3.889 | 16.480 | 20.148 | 24.253 | 3 | 2009-2015 |
| Age-standardized mortality rate per 100K residents | Guatemala | 624.673 | 78.175 | 573.660 | 585.684 | 714.674 | 3 | 2009-2015 |
| Gini index | Guatemala | 0.390 | NA | 0.390 | 0.390 | 0.390 | 2 | 2017 |
| Segregation | Guatemala | 0.550 | 0.068 | 0.473 | 0.580 | 0.598 | 3 | 2002 |
| Population | Guatemala | 911,459 | 1,332,027 | 111,201 | 174,052 | 2,449,123 | 3 | 2009-2015 |
| Population >65 (%) | Guatemala | 4.897 | 0.350 | 4.600 | 4.809 | 5.284 | 3 | 2009-2015 |
| Population density per km2 | Guatemala | 9,432 | 3,265 | 5,894 | 10,077 | 12,326 | 3 | 2009-2015 |
| Poverty (%) | Guatemala | 25.000 | 10.086 | 13.400 | 29.900 | 31.700 | 3 | 2002 |
| Living conditions score | Guatemala | -0.732 | 0.500 | -1.300 | -0.540 | -0.357 | 3 | 2002 |
| Households with piped water inside the dwelling (%) | Guatemala | 71.963 | 4.927 | 66.331 | 74.081 | 75.478 | 2 | 2002 |
| Overcrowding: households with more than 3 people per room (%) | Guatemala | 20.824 | 4.787 | 17.171 | 19.056 | 26.244 | 3 | 2002 |
| 15-17 yo attending school (%) | Guatemala | 62.411 | 2.958 | 59.203 | 63.001 | 65.031 | 3 | 2002 |
| Secondary education (%) | Guatemala | 21.951 | 6.545 | 14.433 | 25.053 | 26.368 | 3 | 2002 |
|  |  |  |  |  |  |  |  |  |
| Annual deaths | Mexico | 43,400 | 121,188 | 4,270 | 20,070 | 1,136,469 | 92 | 2005-2015 |
| Annual temperature range (°C) | Mexico | 17.024 | 6.801 | 5.481 | 15.203 | 34.251 | 92 | 1996-2015 |
| Daily temperature (°C) | Mexico | 20.588 | 4.023 | 12.212 | 20.301 | 26.866 | 92 | 1996-2015 |
| Minimum Mortality Temperature (°C) | Mexico | 23.107 | 3.435 | 16.458 | 23.277 | 33.467 | 92 | 2005-2015 |
| Age-standardized mortality rate per 100K residents | Mexico | 502.010 | 72.626 | 277.378 | 499.588 | 693.029 | 92 | 2005-2015 |
| Gini index | Mexico | 0.449 | 0.059 | 0.292 | 0.447 | 0.656 | 92 | 2010 |
| Segregation | Mexico | 0.142 | 0.031 | 0.085 | 0.137 | 0.223 | 92 | 2010 |
| Population | Mexico | 674,757 | 2,075,386 | 53,695 | 246,518 | 19,334,722 | 92 | 2005-2015 |
| Population >65 (%) | Mexico | 5.654 | 1.203 | 1.445 | 5.579 | 5.654 | 92 | 2005-2015 |
| Population density per km2 | Mexico | 6,393 | 2,399 | 2,446 | 6,004 | 23,200 | 92 | 2005-2015 |
| Poverty (%) | Mexico | 47.101 | 9.918 | 27.700 | 46.250 | 71.100 | 92 | 2010 |
| Living conditions score | Mexico | 0.761 | 1.260 | -3.453 | 1.105 | 2.250 | 92 | 2010 |
| Households with piped water inside the dwelling (%) | Mexico | 78.175 | 14.399 | 33.413 | 80.934 | 96.057 | 92 | 2010 |
| Overcrowding: households with more than 3 people per room (%) | Mexico | 9.932 | 3.987 | 2.960 | 9.178 | 20.722 | 92 | 2010 |
| 15-17 yo attending school (%) | Mexico | 69.696 | 7.038 | 45.749 | 70.869 | 80.391 | 92 | 2010 |
| Secondary education (%) | Mexico | 31.955 | 7.412 | 13.037 | 33.223 | 46.333 | 92 | 2010 |
| Annual deaths | Panama | 13,843 | 16,180 | 4,267 | 4,737 | 32,524 | 3 | 2012-2015 |
| Annual temperature range (°C) | Panama | 4.512 | 1.149 | 3.630 | 4.095 | 5.811 | 3 | 1996-2015 |
| Daily temperature (°C) | Panama | 25.316 | 0.416 | 24.836 | 25.542 | 25.571 | 3 | 1996-2015 |
| Minimum Mortality Temperature (°C) | Panama | 26.069 | 1.075 | 25.409 | 25.490 | 27.309 | 3 | 2012-2015 |
| Age-standardized mortality rate per 100K residents | Panama | 485.284 | 44.507 | 435.031 | 501.093 | 519.728 | 3 | 2012-2015 |
| Gini index | Panama | 0.495 | 0.034 | 0.460 | 0.497 | 0.528 | 3 | 2010 |
| Segregation | Panama | 0.141 | 0.028 | 0.123 | 0.127 | 0.173 | 3 | 2010 |
| Population | Panama | 493,155 | 630,684 | 109,027 | 149,404 | 1,221,033 | 3 | 2012-2015 |
| Population >65 (%) | Panama | 7.028 | 1.554 | 5.567 | 6.856 | 7.028 | 3 | 2012-2015 |
| Population density per km2 | Panama | 6,409 | 1,736 | 4,614 | 6,535 | 8,079 | 3 | 2012-2015 |
| Poverty (%) | Panama | 11.633 | 0.321 | 11.400 | 11.500 | 12.000 | 3 | 2015 |
| Living conditions score | Panama | -0.942 | 1.172 | -2.094 | -0.983 | 0.249 | 3 | 2010 |
| Households with piped water inside the dwelling (%) | Panama | 81.905 | 4.025 | 77.554 | 82.666 | 85.495 | 3 | 2010 |
| Overcrowding: households with more than 3 people per room (%) | Panama | 4.336 | 0.947 | 3.365 | 4.385 | 5.257 | 3 | 2010 |
| 15-17 yo attending school (%) | Panama | 86.838 | 0.752 | 86.335 | 86.476 | 87.703 | 3 | 2010 |
| Secondary education (%) | Panama | 52.081 | 1.893 | 50.871 | 51.109 | 54.262 | 3 | 2010 |
|  |  |  |  |  |  |  |  |  |
| Annual deaths | Peru | 21754 | 54238 | 2518 | 8337 | 261923 | 22 | 2008-2015 |
| Annual temperature range (°C) | Peru | 7.249 | 1.505 | 4.248 | 7.315 | 9.234 | 22 | 1996-2015 |
| Daily temperature (°C) | Peru | 17.655 | 6.183 | 7.855 | 19.534 | 25.712 | 22 | 1996-2015 |
| Minimum Mortality Temperature (°C) |  | 19.486 | 5.438 | 10.118 | 21.205 | 25.666 |  | 2008-2015 |
| Age-standardized mortality rate per 100K residents | Peru | 3.554 | 0.924 | 1.282 | 3.807 | 4.562 | 22 | 2008-2015 |
| Gini index | Peru | 0.400 | NA | 0.400 | 0.400 | 0.400 | 1 | 2016 |
| Segregation | Peru | NA | NA | NA | NA | NA | NA | NA |
| Population | Peru | 690,570 | 1,837,560 | 51,898 | 256,233 | 8,866,886 | 22 | 2008-2015 |
| Population >65 (%) | Peru | 5.301 | 1.156 | 3.211 | 5.354 | 7.205 | 22 | 2008-2015 |
| Population density per km2 | Peru | 11,754 | 2,787 | 7,822 | 11,502 | 17,273 | 22 | 2008-2015 |
| Poverty (%) | Peru | 23.109 | 9.734 | 9.300 | 21.400 | 45.100 | 22 | 2009 |
| Living conditions score | Peru | -1.220 | 1.200 | -4.299 | -1.226 | 0.805 | 22 | 2007 |
| Households with piped water inside the dwelling (%) | Peru | 68.583 | 11.307 | 36.362 | 71.769 | 82.254 | 22 | 2007 |
| Overcrowding: households with more than 3 people per room (%) | Peru | 12.284 | 3.833 | 5.752 | 10.782 | 20.482 | 22 | 2007 |
| 15-17 yo attending school (%) | Peru | 80.377 | 4.975 | 71.510 | 80.295 | 87.318 | 22 | 2007 |
| Secondary education (%) | Peru | 59.195 | 7.280 | 49.179 | 58.382 | 72.988 | 22 | 2007 |

Table S2. Differences in excess death fractions (EDF) of all-cause mortality associated with cold and hot temperatures by levels of socioeconomic and demographic characteristics of Latin American cities. Cold temperatures are defined as those below the minimum mortality temperature. Hot temperatures are defined as those above the minimum mortality temperature. Point estimates and 95% confidence intervals are obtained from the random effects meta-regressions that include a socioeconomic/demographic indicator, mean daily temperature, mean annual temperature range, climate zone, and country group. Separate meta-regressions were fitted for each indicator. The socioeconomic/demographic characteristics were classified as low, medium, and high according to the tertiles of their distribution. The reference category for each effect modifier are cities with desirable levels of the indicator (e.g., low poverty, high living conditions score, etc.). In the case of population, population density, and % built-up area, the reference are cities with low (bottom tertile) absolute values of these characteristics. The analysis is based on 325 cities for all variables except poverty (n=319 cities), Gini index (n=296), and isolation index (n=303). Refer to Table 1 in main text for variables’ definition.

| Variable | EDF type | Estimate | Std. Error | 95% CI Lower Bound | 95% CI Upper Bound |
| --- | --- | --- | --- | --- | --- |
| Population, medium | cold | -0.363 | 1.069 | -2.458 | 1.731 |
| Population, high | cold | 0.470 | 1.079 | -1.644 | 2.584 |
| Population density, medium | cold | -0.191 | 1.103 | -2.353 | 1.972 |
| Population density, high | cold | 2.574 | 1.411 | -0.192 | 5.340 |
| Built-up area (%), medium | cold | 1.920 | 1.084 | -0.205 | 4.045 |
| Built-up area (%), high | cold | 0.709 | 1.134 | -1.514 | 2.931 |
| Age-standardized mortality rate, medium | cold | -1.698 | 1.182 | -4.014 | 0.619 |
| Age-standardized mortality rate, high | cold | -1.047 | 1.305 | -3.604 | 1.511 |
| Living conditions score, medium | cold | 0.398 | 1.206 | -1.967 | 2.763 |
| Living conditions score, low | cold | 0.970 | 1.886 | -2.726 | 4.666 |
| Secondary education (%), medium | cold | 1.389 | 1.121 | -0.808 | 3.585 |
| Secondary education (%), high | cold | 0.361 | 1.231 | -2.052 | 2.773 |
| Poverty (%), medium | cold | 1.700 | 1.278 | -0.805 | 4.204 |
| Poverty (%), high | cold | 2.120 | 1.578 | -0.973 | 5.213 |
| Gini index, medium inequality | cold | 2.676 | 1.313 | 0.104 | 5.249 |
| Gini index, high inequality | cold | 3.511 | 1.593 | 0.388 | 6.633 |
| Segregation, medium | cold | 0.768 | 1.228 | -1.639 | 3.175 |
| Segregation, high | cold | 1.332 | 1.553 | -1.711 | 4.375 |
| Population, medium | heat | -0.347 | 0.182 | -0.704 | 0.009 |
| Population, high | heat | -0.214 | 0.179 | -0.565 | 0.136 |
| Population density, medium | heat | -0.080 | 0.183 | -0.439 | 0.279 |
| Population density, high | heat | -0.701 | 0.232 | -1.155 | -0.246 |
| Built-up area (%), medium | heat | -0.124 | 0.184 | -0.485 | 0.237 |
| Built-up area (%), high | heat | -0.259 | 0.188 | -0.627 | 0.109 |
| Age-standardized mortality rate, medium | heat | 0.013 | 0.203 | -0.384 | 0.409 |
| Age-standardized mortality rate, high | heat | 0.243 | 0.226 | -0.200 | 0.686 |
| Living conditions score, medium | heat | -0.183 | 0.201 | -0.578 | 0.212 |
| Living conditions score, low | heat | -0.481 | 0.331 | -1.130 | 0.167 |
| Secondary education (%), medium | heat | -0.017 | 0.201 | -0.410 | 0.376 |
| Secondary education (%), high | heat | 0.112 | 0.214 | -0.307 | 0.531 |
| Poverty (%), medium | heat | -0.527 | 0.259 | -1.035 | -0.019 |
| Poverty (%), high | heat | -1.077 | 0.324 | -1.713 | -0.441 |
| Gini index, medium inequality | heat | -0.574 | 0.302 | -1.165 | 0.018 |
| Gini index, high inequality | heat | -1.146 | 0.375 | -1.880 | -0.411 |


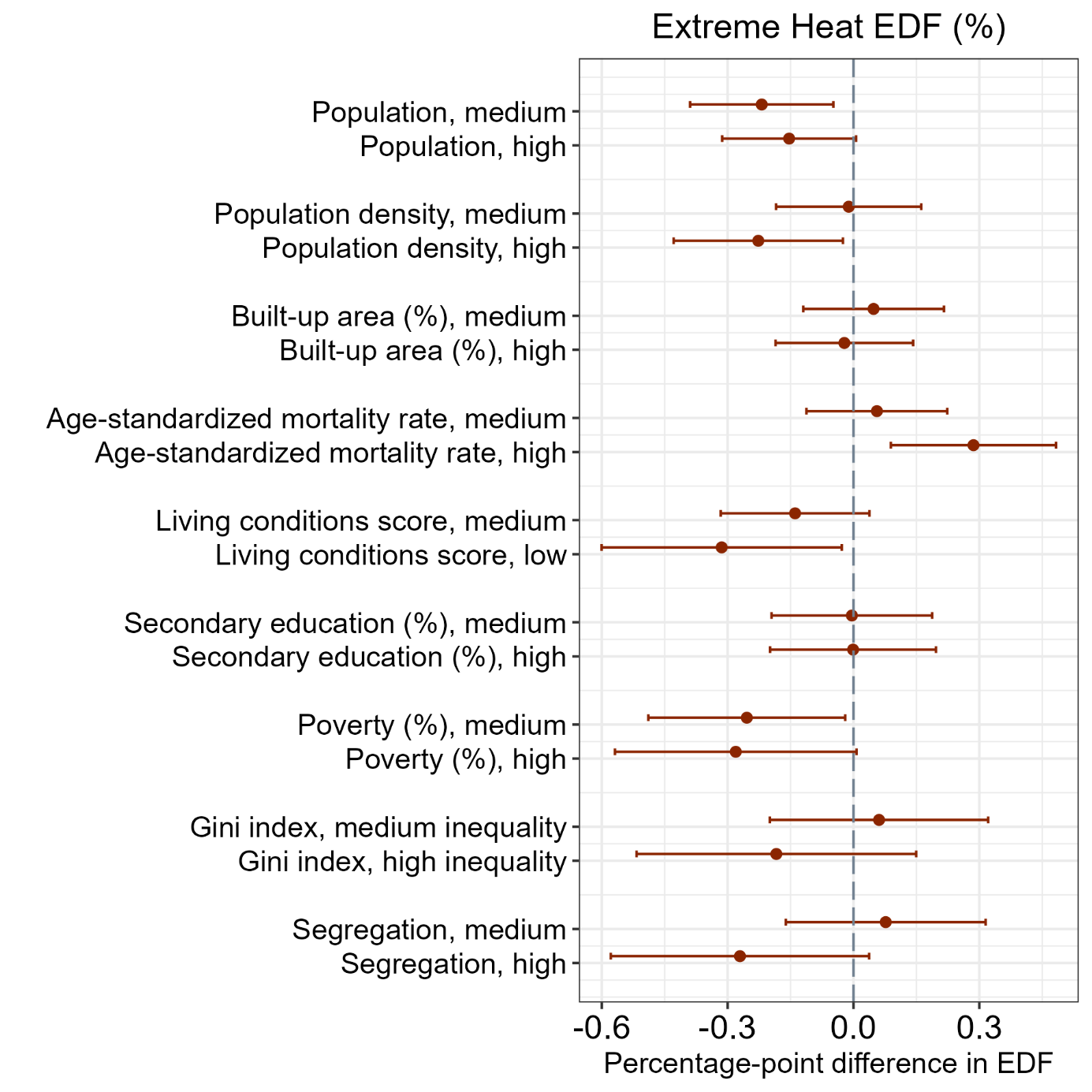

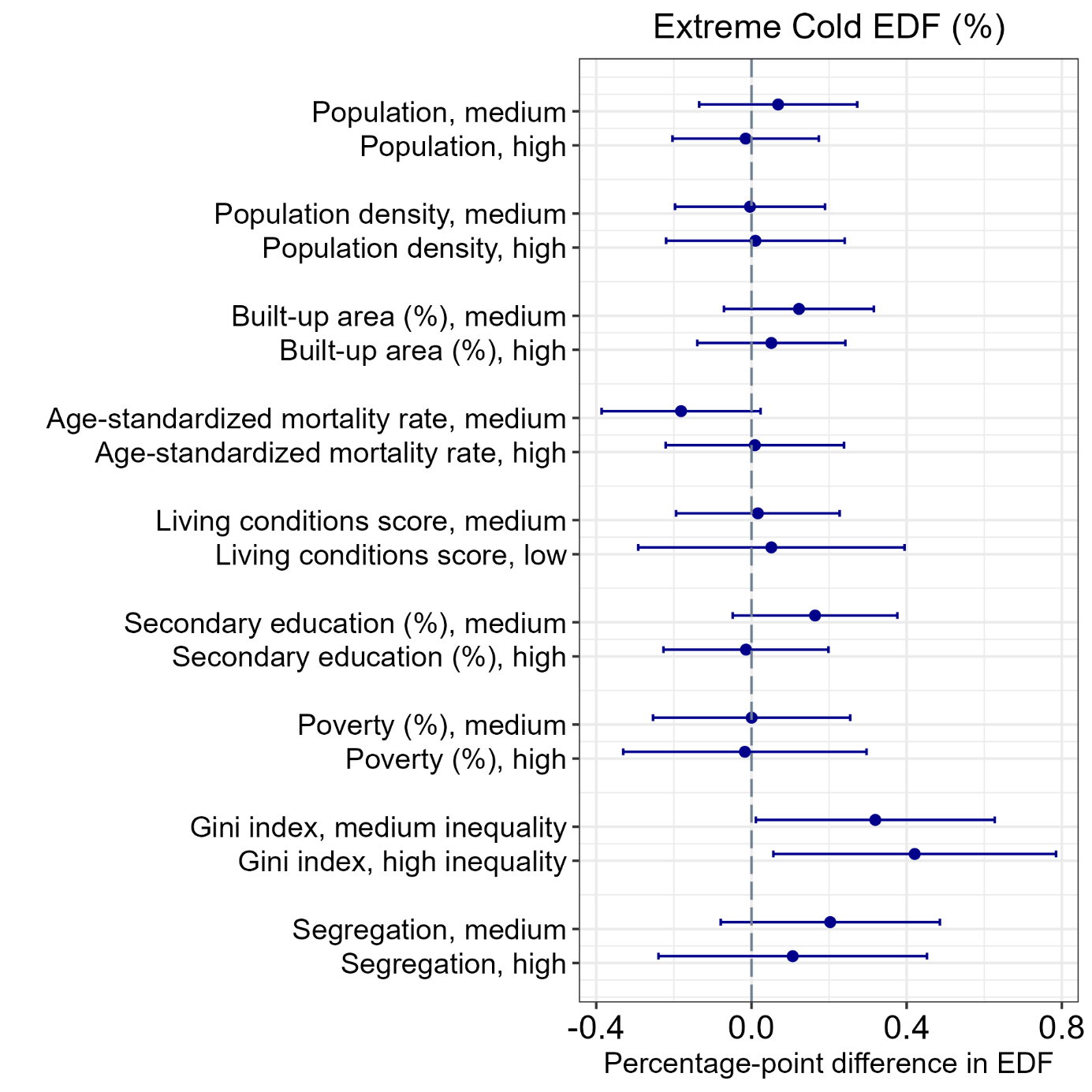


Figure S1. Differences in excess death fractions (EDF) of all-cause mortality associated with extremely cold and extremely hot temperatures by levels of socioeconomic and demographic characteristics of Latin American cities. Extreme cold is defined as temperatures ≤5^th^ percentile of city-specific daily temperatures. Extreme heat is defined as temperatures ≥95^th^ percentile of city-specific daily temperatures. Point estimates and 95% confidence intervals are obtained from the random effects meta-regressions that include a socioeconomic indicator, mean daily temperature, mean annual temperature range, climate zone, and country group. Separate meta-regressions were fitted for each indicator. The socioeconomic/demographic characteristics were classified as low, medium, and high according to the tertiles of their distribution. The reference category for each effect modifier are cities with desirable levels of the indicator (e.g., low poverty, high living conditions score, etc.). In the case of population, population density, and % built-up area, the reference are cities with low (bottom tertile) absolute values of these characteristics. The analysis is based on 325 cities for all variables except poverty (n=319 cities), Gini index (n=296), and isolation index (n=303). Refer to Table 1 for variables’ definition. Supplementary Material Table S3 contains the estimates and confidence intervals shown in the figure.

Table S3. Differences in excess death fractions (EDF) of all-cause mortality associated with extremely cold and extremely hot temperatures by levels of the socioeconomic and demographic characteristics of 325 Latin American cities. Refer to Figure S1 above for technical details.

| Variable | EDF type | Estimate | Std. Error | 95% CI Lower Bound | 95% CI Upper Bound |
| --- | --- | --- | --- | --- | --- |
| Population, medium | extreme cold | 0.069 | 0.104 | -0.135 | 0.272 |
| Population, high | extreme cold | -0.015 | 0.096 | -0.204 | 0.174 |
| Population density, medium | extreme cold | -0.004 | 0.099 | -0.197 | 0.189 |
| Population density, high | extreme cold | 0.010 | 0.117 | -0.220 | 0.240 |
| Built-up area (%), medium | extreme cold | 0.122 | 0.099 | -0.071 | 0.315 |
| Built-up area (%), high | extreme cold | 0.051 | 0.097 | -0.140 | 0.242 |
| Age-standardized mortality rate, medium | extreme cold | -0.181 | 0.105 | -0.386 | 0.024 |
| Age-standardized mortality rate, high | extreme cold | 0.009 | 0.117 | -0.221 | 0.239 |
| Living conditions score, medium | extreme cold | 0.016 | 0.108 | -0.194 | 0.227 |
| Living conditions score, low | extreme cold | 0.051 | 0.175 | -0.292 | 0.395 |
| Secondary education (%), medium | extreme cold | 0.164 | 0.108 | -0.048 | 0.376 |
| Secondary education (%), high | extreme cold | -0.014 | 0.108 | -0.227 | 0.199 |
| Poverty (%), medium | extreme cold | 0.000 | 0.130 | -0.254 | 0.255 |
| Poverty (%), high | extreme cold | -0.017 | 0.160 | -0.331 | 0.297 |
| Gini index, medium inequality | extreme cold | 0.319 | 0.157 | 0.011 | 0.627 |
| Gini index, high inequality | extreme cold | 0.421 | 0.186 | 0.056 | 0.785 |
| Segregation, medium | extreme cold | 0.203 | 0.144 | -0.079 | 0.485 |
| Segregation, high | extreme cold | 0.106 | 0.177 | -0.240 | 0.452 |
| Population, medium | extreme heat | -0.219 | 0.087 | -0.389 | -0.048 |
| Population, high | extreme heat | -0.153 | 0.081 | -0.313 | 0.006 |
| Population density, medium | extreme heat | -0.011 | 0.088 | -0.184 | 0.162 |
| Population density, high | extreme heat | -0.227 | 0.103 | -0.429 | -0.025 |
| Built-up area (%), medium | extreme heat | 0.048 | 0.086 | -0.120 | 0.216 |
| Built-up area (%), high | extreme heat | -0.022 | 0.084 | -0.186 | 0.142 |
| Age-standardized mortality rate, medium | extreme heat | 0.056 | 0.086 | -0.112 | 0.224 |
| Age-standardized mortality rate, high | extreme heat | 0.286 | 0.100 | 0.089 | 0.483 |
| Living conditions score, medium | extreme heat | -0.139 | 0.090 | -0.316 | 0.038 |
| Living conditions score, low | extreme heat | -0.314 | 0.146 | -0.600 | -0.028 |
| Secondary education (%), medium | extreme heat | -0.004 | 0.098 | -0.195 | 0.188 |
| Secondary education (%), high | extreme heat | -0.001 | 0.101 | -0.199 | 0.197 |
| Poverty (%), medium | extreme heat | -0.254 | 0.120 | -0.489 | -0.020 |
| Poverty (%), high | extreme heat | -0.281 | 0.147 | -0.569 | 0.008 |
| Gini index, medium inequality | extreme heat | 0.061 | 0.133 | -0.199 | 0.321 |
| Gini index, high inequality | extreme heat | -0.184 | 0.170 | -0.517 | 0.149 |

Table S4. Interaction relative risks (IRRs) of all-cause mortality per 1°C more extreme cold and extreme hot temperatures by levels of the socioeconomic characteristics of Latin American cities. The IRRs represent proportional difference in RR per 1°C associated with the given characteristic. RR for extreme cold was computed by dividing the difference in log-relative risk of mortality between temperatures at the 1^st^ and 5^th^ percentile of the city-specific daily mean temperature distribution by the difference in degrees Celsius between the 1^st^ percentile and 5^th^ percentile of the temperature distribution, and exponentiating the quotient. RR for heat was analogously obtained as the difference between the log-relative risk of mortality at the 99^th^ and 95^th^ percentile of the city-specific observed distribution of daily temperatures divided by the difference in degrees Celsius between the 99^th^ percentile and 95^th^ percentile of the temperature distribution, and exponentiating the quotient. For extreme cold, the IRR results can be interpreted as a difference in the relative risk of mortality associated with a 1°C decrease in mean daily temperature below the 5^th^ percentile of the temperature distribution. For extreme heat, the IRR results present an estimated change in the relative risk of mortality associated with a 1°C increase in daily mean temperature above the 95^th^ percentile of the temperature distribution. Point estimates and 95% confidence intervals are obtained from the random effects meta-regressions that include a socioeconomic indicator, mean daily temperature, mean annual temperature range, climate zone, and country group. Separate meta-regressions were fitted for each socioeconomic indicator. The reference category for each socioeconomic effect modifier are cities with desirable levels of the indicator (e.g., low poverty, high living conditions score, etc.). In the case of population, population density, and % urban area, the reference are cities with low absolute values (bottom tertile) of these characteristics. The analysis is based on 325 cities for all variables except poverty (n=319 cities), Gini index (n=296), and segregation (n=303). Refer to Table 1 for variables’ definition.

| Variable | IRR type | IRR Estimate | Std. Error | 95% CI Lower Bound | 95% CI Upper Bound |
| --- | --- | --- | --- | --- | --- |
| Population, medium | extreme cold | 1.014 | 0.997 | 1.031 | 1.014 |
| Population, high | extreme cold | 1.005 | 0.990 | 1.020 | 1.005 |
| Population density, medium | extreme cold | 1.002 | 0.990 | 1.015 | 1.002 |
| Population density, high | extreme cold | 1.003 | 0.986 | 1.021 | 1.003 |
| Built-up area (%), medium | extreme cold | 0.999 | 0.984 | 1.015 | 0.999 |
| Built-up area (%), high | extreme cold | 1.002 | 0.988 | 1.016 | 1.002 |
| Age-standardized mortality rate, medium | extreme cold | 0.992 | 0.977 | 1.007 | 0.992 |
| Age-standardized mortality rate, high | extreme cold | 1.004 | 0.988 | 1.021 | 1.004 |
| Living conditions score, medium | extreme cold | 1.003 | 0.987 | 1.020 | 1.003 |
| Living conditions score, low | extreme cold | 0.990 | 0.966 | 1.015 | 0.990 |
| Secondary education (%), medium | extreme cold | 1.000 | 0.985 | 1.016 | 1.000 |
| Secondary education (%), high | extreme cold | 0.993 | 0.976 | 1.010 | 0.993 |
| Poverty (%), medium | extreme cold | 0.999 | 0.983 | 1.015 | 0.999 |
| Poverty (%), high | extreme cold | 0.982 | 0.961 | 1.005 | 0.982 |
| Gini index, medium inequality | extreme cold | 1.010 | 0.993 | 1.028 | 1.010 |
| Gini index, high inequality | extreme cold | 1.017 | 0.996 | 1.039 | 1.017 |
| Segregation, medium | extreme cold | 1.012 | 0.994 | 1.031 | 1.012 |
| Segregation, high | extreme cold | 1.016 | 0.992 | 1.041 | 1.016 |
| Population, medium | extreme heat | 0.993 | 0.965 | 1.021 | 0.993 |
| Population, high | extreme heat | 0.998 | 0.973 | 1.023 | 0.998 |
| Population density, medium | extreme heat | 1.014 | 0.993 | 1.035 | 1.014 |
| Population density, high | extreme heat | 0.994 | 0.967 | 1.022 | 0.994 |
| Built-up area (%), medium | extreme heat | 0.995 | 0.971 | 1.020 | 0.995 |
| Built-up area (%), high | extreme heat | 1.010 | 0.988 | 1.033 | 1.010 |
| Age-standardized mortality rate, medium | extreme heat | 0.992 | 0.977 | 1.007 | 0.992 |
| Age-standardized mortality rate, high | extreme heat | 1.004 | 0.988 | 1.021 | 1.004 |
| Living conditions score, medium | extreme heat | 1.001 | 0.974 | 1.029 | 1.001 |
| Living conditions score, low | extreme heat | 0.985 | 0.944 | 1.027 | 0.985 |
| Secondary education (%), medium | extreme heat | 0.995 | 0.970 | 1.020 | 0.995 |
| Secondary education (%), high | extreme heat | 0.985 | 0.958 | 1.012 | 0.985 |
| Poverty (%), medium | extreme heat | 1.000 | 0.976 | 1.024 | 1.000 |
| Poverty (%), high | extreme heat | 0.980 | 0.945 | 1.016 | 0.980 |
| Gini index, medium inequality | extreme heat | 1.020 | 0.990 | 1.052 | 1.020 |
| Gini index, high inequality | extreme heat | 1.011 | 0.975 | 1.049 | 1.011 |


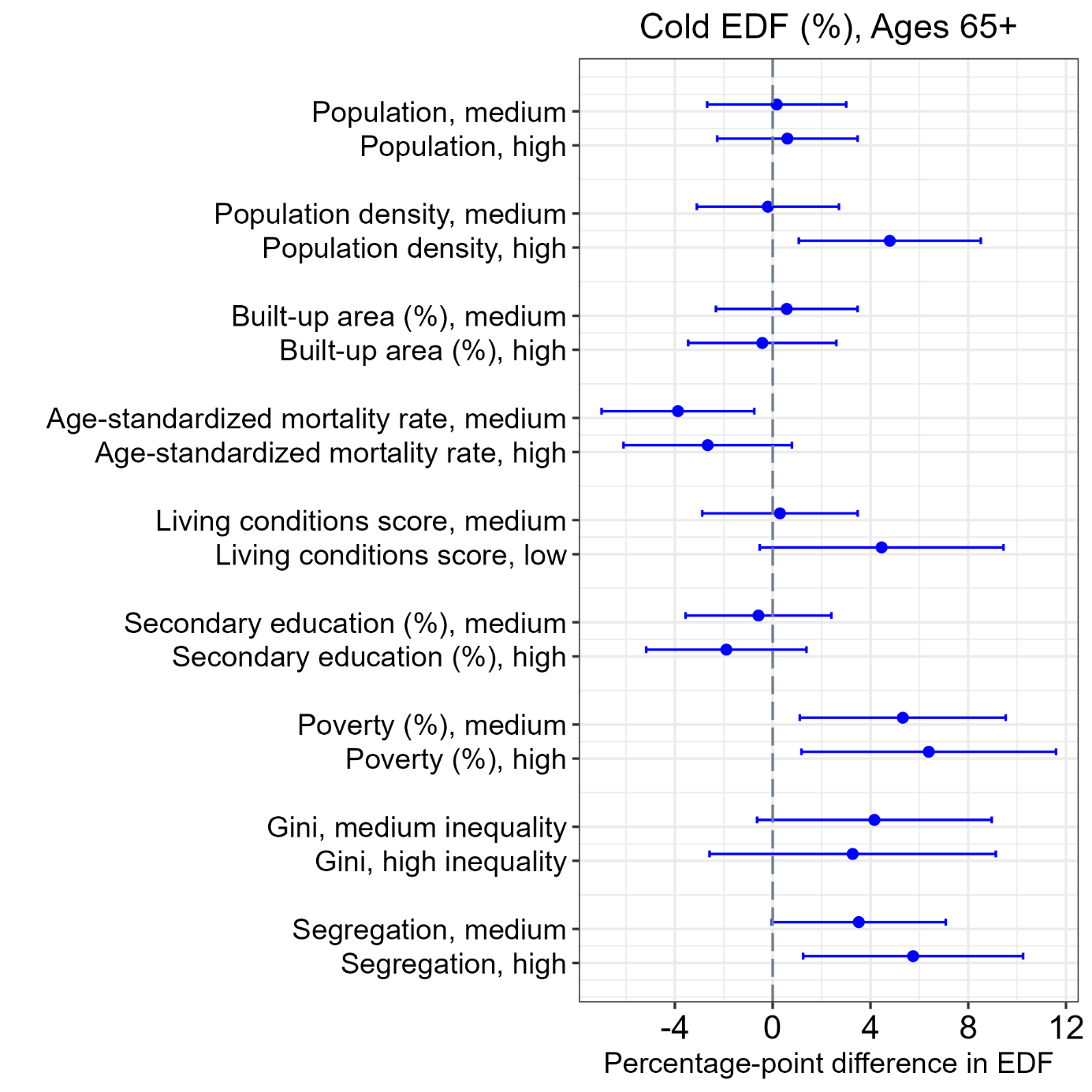

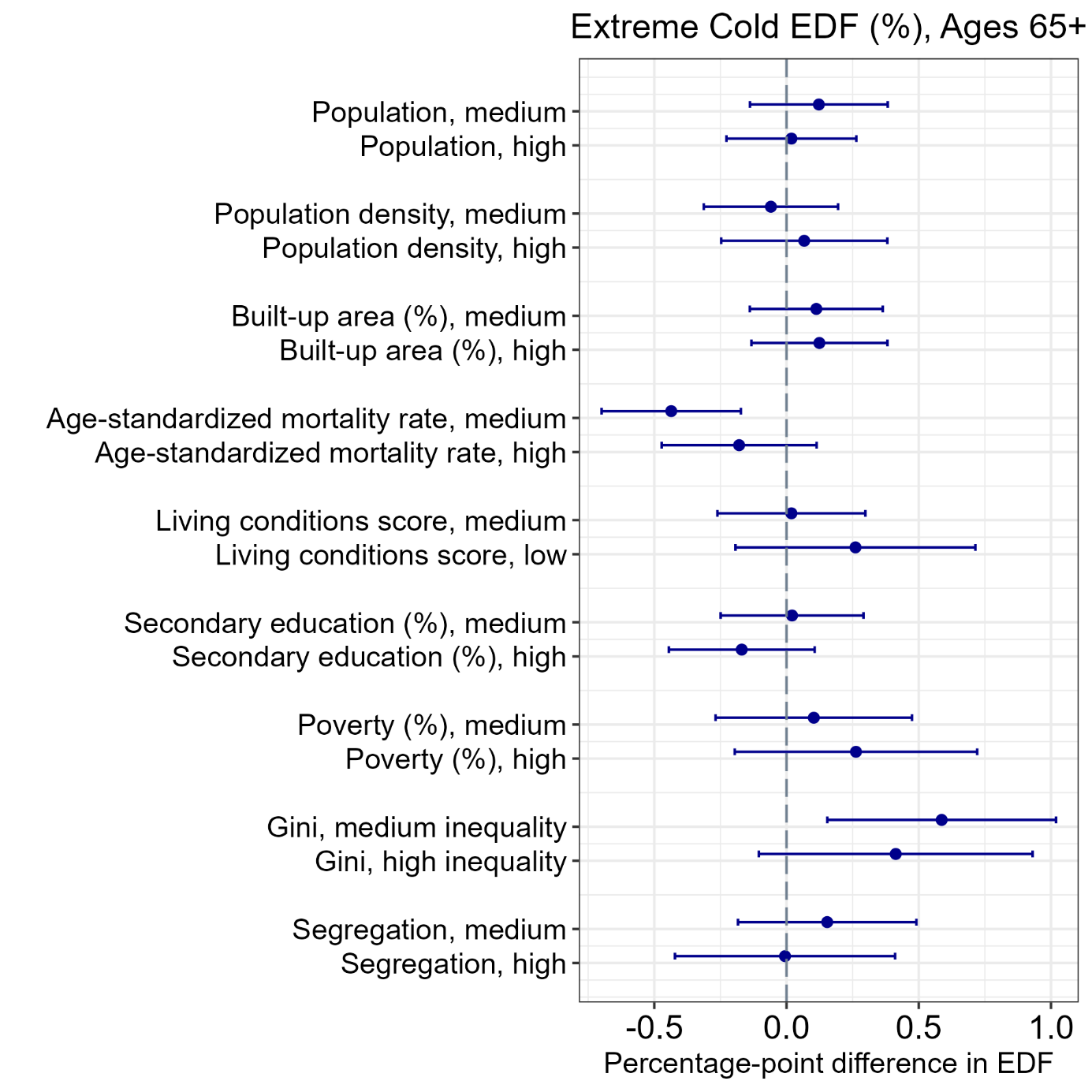


Figure S2. Differences in excess death fractions (EDF) associated with cold and extremely cold temperatures for population 65 years and older by levels of the socioeconomic and demographic characteristics of Latin American cities. Cold temperatures are defined as those below the minimum mortality temperature. Extreme cold is defined as temperatures ≤5th percentile of city-specific daily temperatures. Point estimates and 95% confidence intervals are obtained from the random effects meta-regressions that include a socioeconomic indicator, mean daily temperature, mean annual temperature range, climate zone, and country group. Separate meta-regressions were fitted for each indicator. The socioeconomic characteristics were classified as low, medium, and high according to the tertiles of their distribution. The reference category for each effect modifier are cities with desirable levels of the indicator (e.g., low poverty, high living conditions score, etc.). In the case of population, population density, and % built-up area, the reference are cities with low (bottom tertile) absolute values of these characteristics. The analysis is based on 325 cities for all variables except poverty (n=319 cities), Gini index (n=296), and isolation index (n=303). Refer to Table 1 in main text for variables’ definition. Supplementary Material Table S5 contains the estimates and confidence intervals shown in the figure.


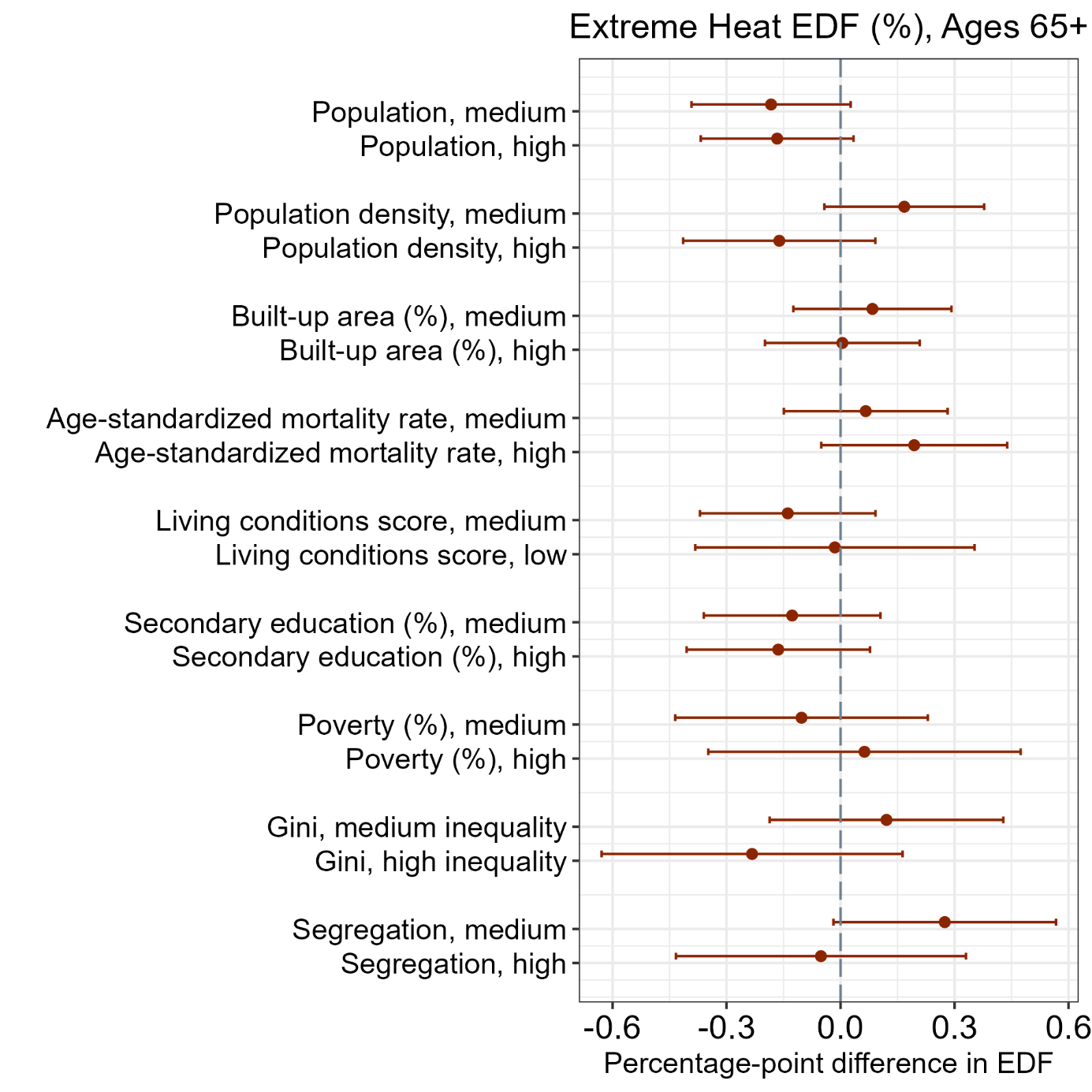

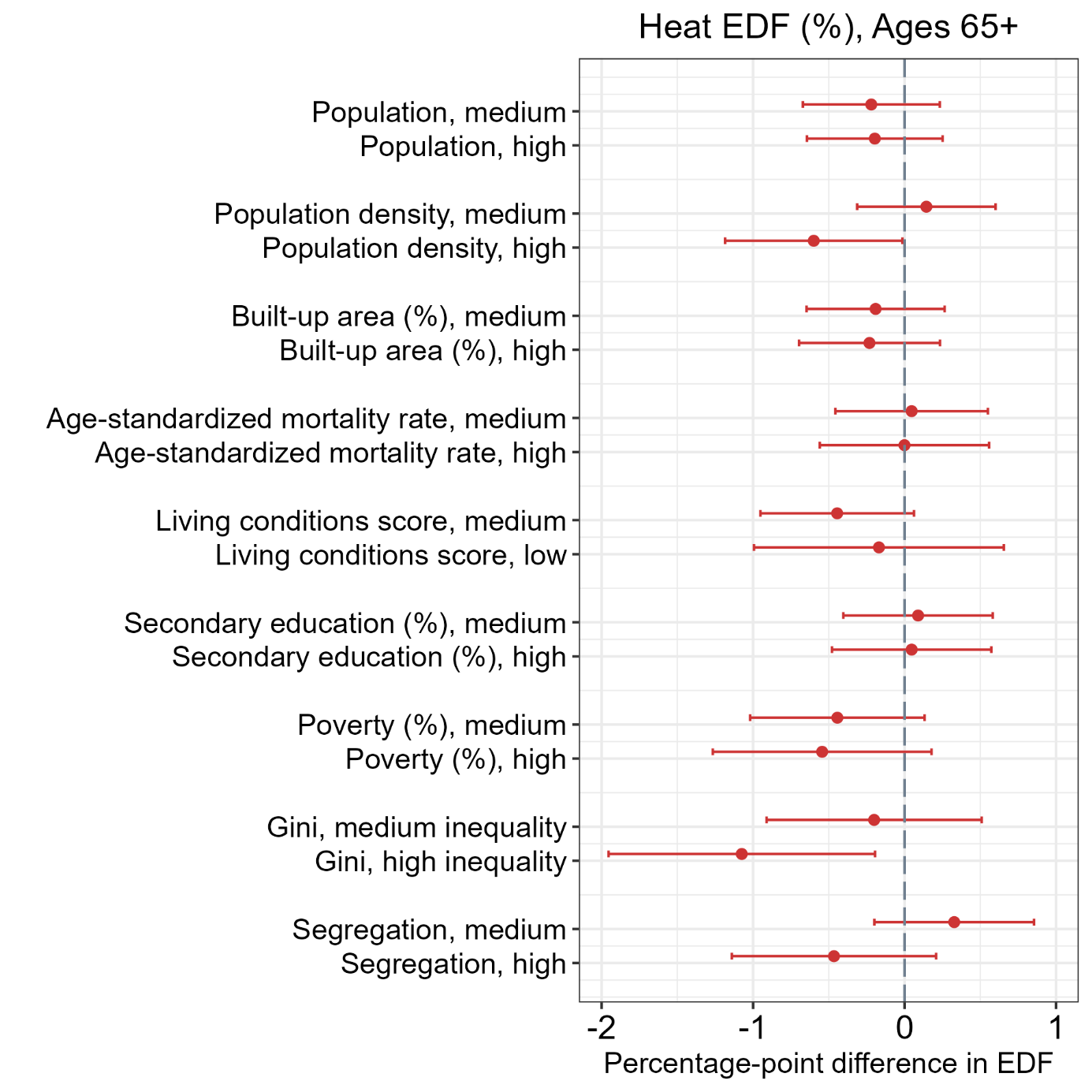


Figure S3. Differences in excess death fractions (EDF) associated with hot and extremely hot temperatures for population 65 years and older by levels of the socioeconomic and demographic characteristics of Latin American cities. Hot temperatures are defined as those above the minimum mortality temperature. Extreme heat is defined as temperatures ≥95^th^ percentile of city-specific daily temperatures. Point estimates and 95% confidence intervals are obtained from the random effects meta-regressions that include a socioeconomic indicator, mean daily temperature, mean annual temperature range, and country group. Separate meta-regressions were fitted for each indicator. The socioeconomic characteristics were classified as low, medium, and high according to the tertiles of their distribution. The reference category for each effect modifier are cities with desirable levels of the indicator (e.g., low unemployment, high living conditions score, etc.). In the case of population, population density, and % built-up area, the reference are cities with low (bottom tertile) absolute values of these characteristics. The analysis is based on 325 cities for all variables except poverty (n=319 cities), Gini index (n=296), and isolation index (n=303). Refer to Table 1 in main text for variables’ definition. Supplementary Material Table S5 contains the estimates and confidence intervals shown in the figure.


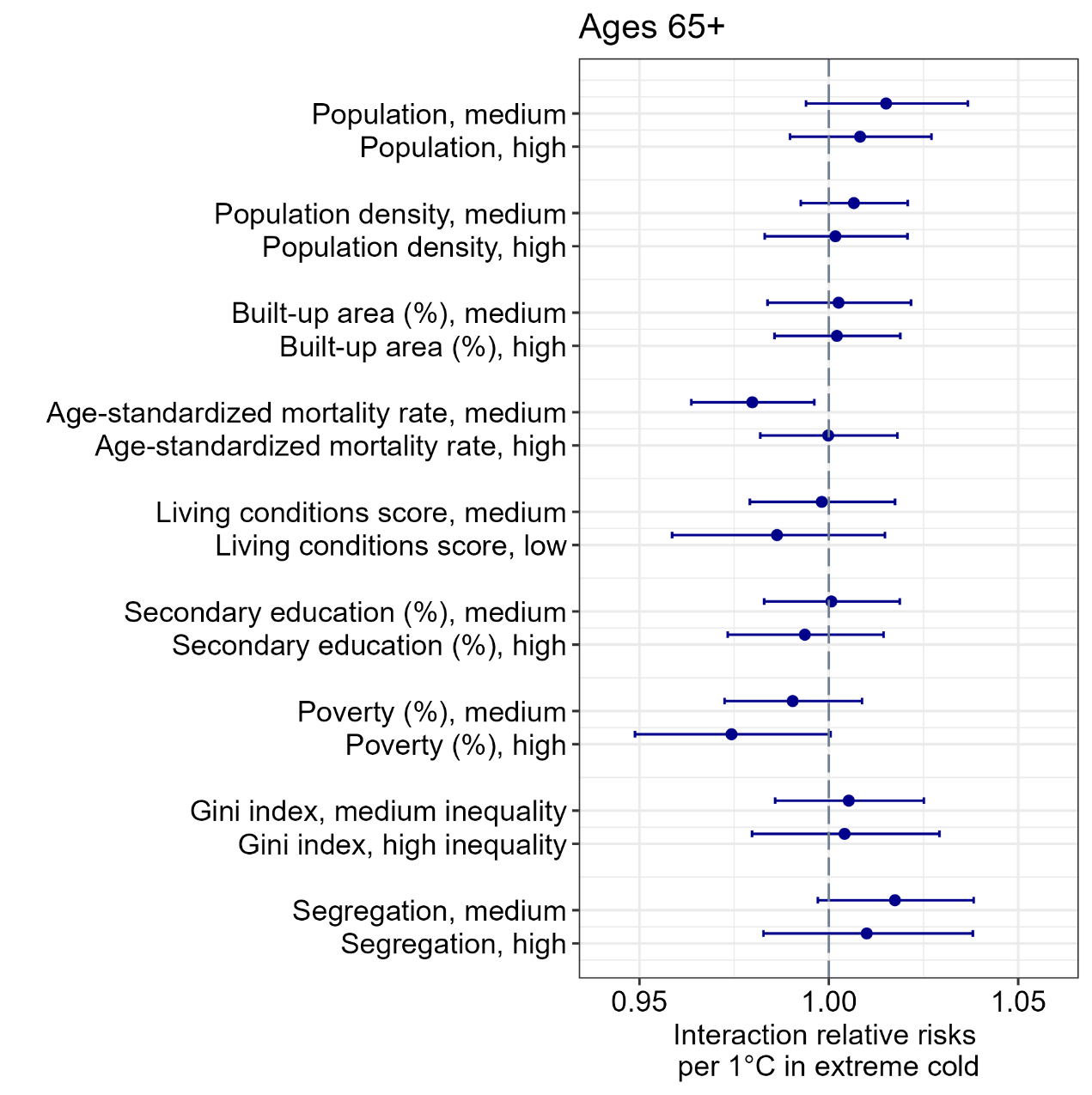

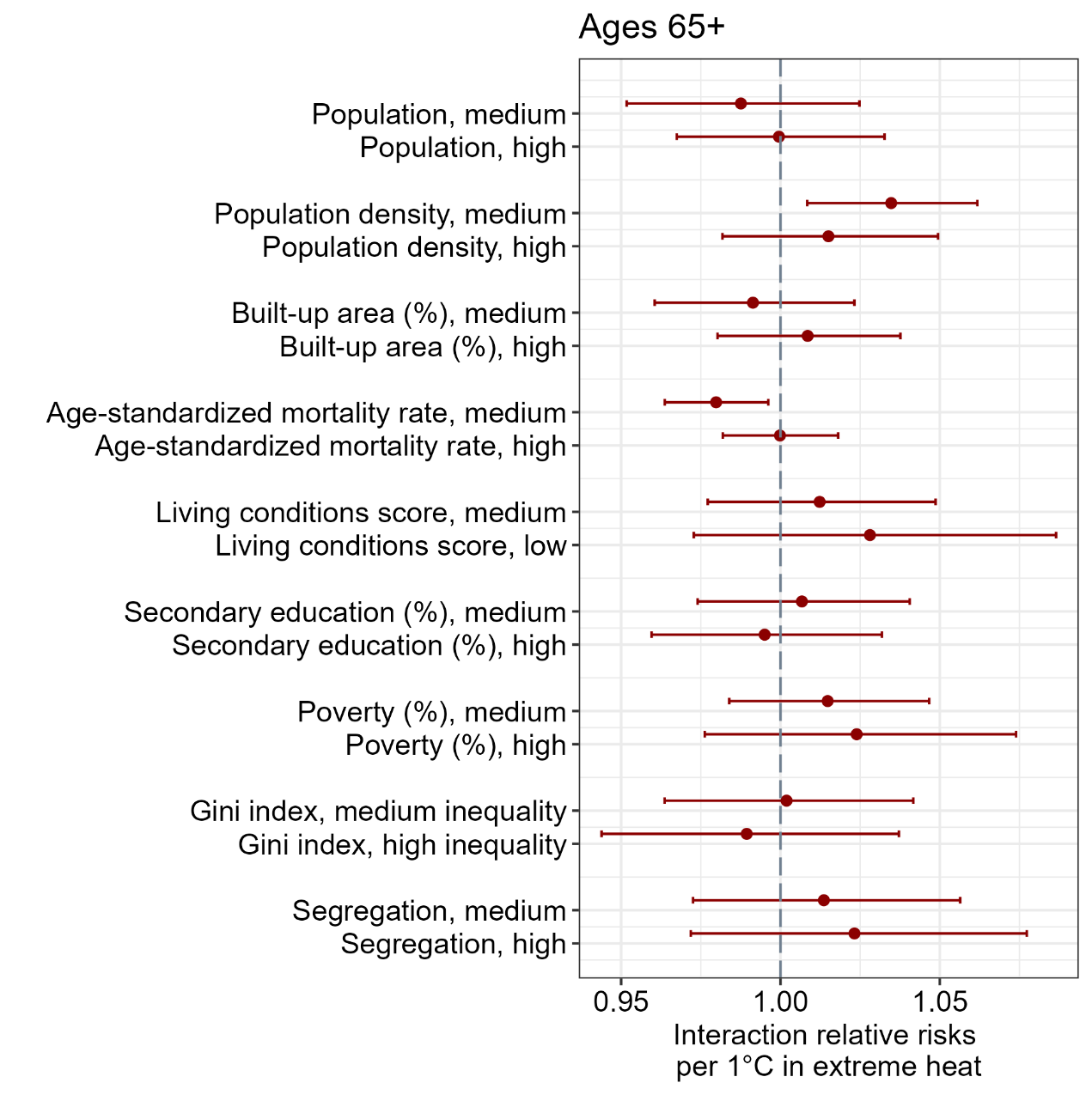


Figure S4. Interaction relative risks (IRRs) of all-cause mortality per 1°C more extreme cold and extreme hot temperatures among the population 65 years and older by levels of the socioeconomic and demographic characteristics of Latin American cities. Table S6 contains technical details and numerical estimates presented in the figure.


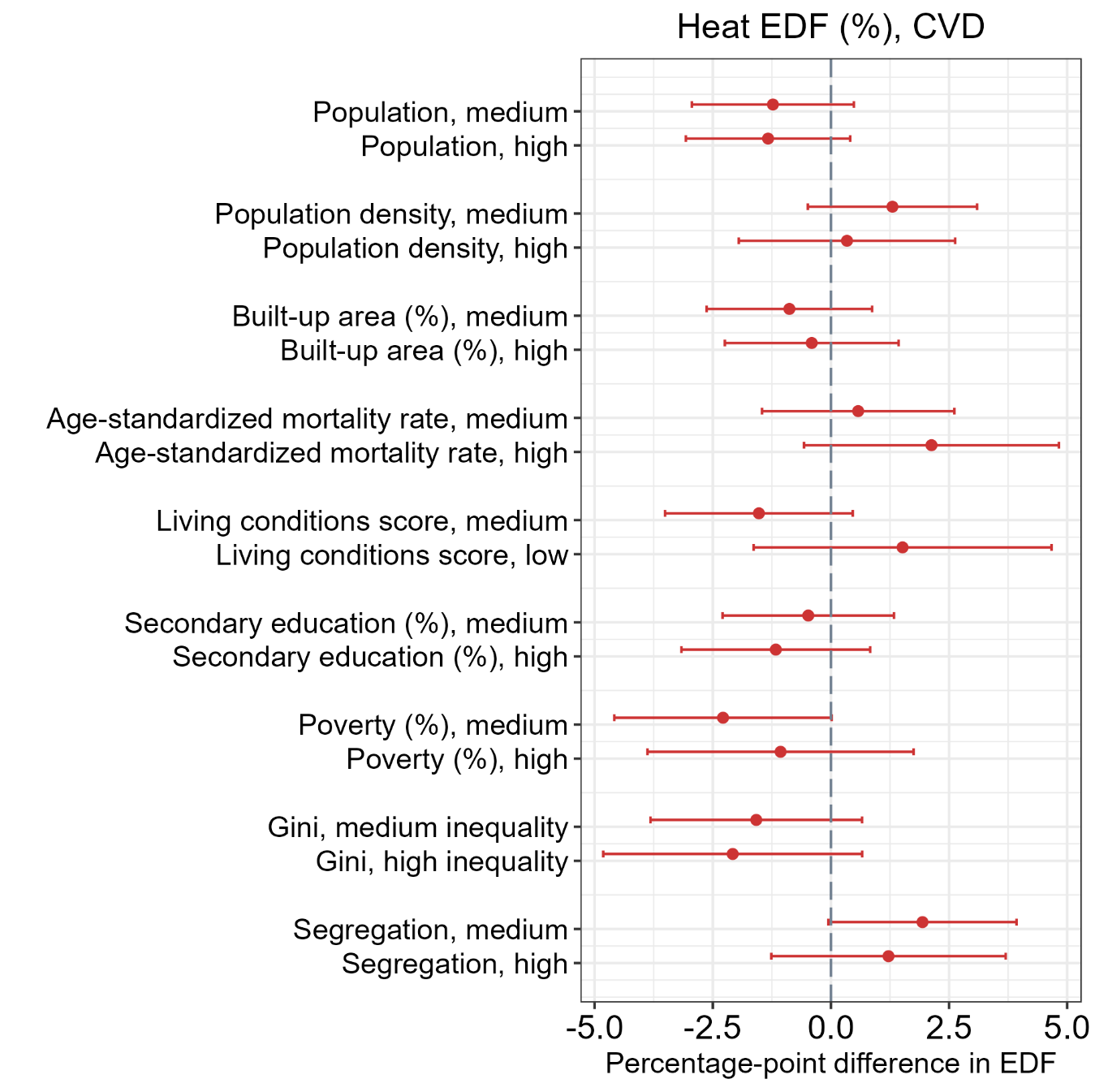

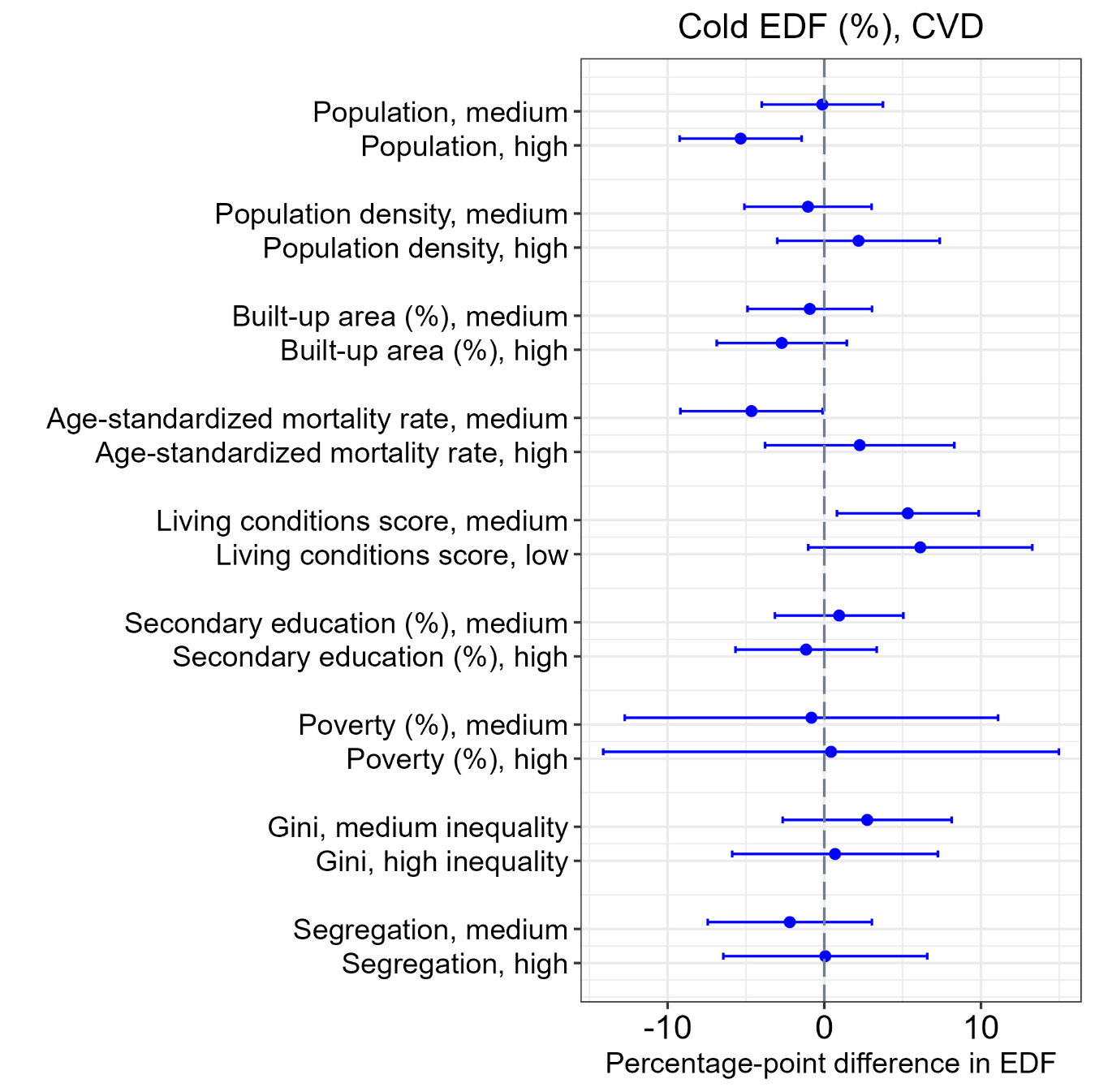


Figure S5. Differences in excess death fractions (EDF) of cardiovascular mortality associated with cold and hot temperatures by levels of the socioeconomic and demographic characteristics of Latin American cities. Table S7 contains technical details and numerical estimates presented in the figure.

Table S5. Differences in excess death fractions (EDF) due to cold, hot, extremely cold, and extremely hot temperatures among the population ages 65+ by levels of the socioeconomic and demographic characteristics of Latin American cities. Refer to Table S2 for technical details.

| Variable | EDF type | Estimate | Std. Error | 95% CI Lower Bound | 95% CI Upper Bound |
| --- | --- | --- | --- | --- | --- |
| Population, medium | cold | 0.16 | 1.45 | -2.68 | 3.01 |
| Population, high | cold | 0.6 | 1.47 | -2.27 | 3.47 |
| Population density, medium | cold | -0.2 | 1.48 | -3.1 | 2.71 |
| Population density, high | cold | 4.79 | 1.9 | 1.07 | 8.51 |
| Built-up area (%), medium | cold | 0.58 | 1.48 | -2.32 | 3.47 |
| Built-up area (%), high | cold | -0.42 | 1.55 | -3.46 | 2.61 |
| Age-standardized mortality rate, medium | cold | -3.87 | 1.59 | -7 | -0.75 |
| Age-standardized mortality rate, high | cold | -2.66 | 1.76 | -6.11 | 0.79 |
| Living conditions score, medium | cold | 0.3 | 1.62 | -2.88 | 3.48 |
| Living conditions score, low | cold | 4.46 | 2.54 | -0.53 | 9.44 |
| Secondary education (%), medium | cold | -0.58 | 1.52 | -3.56 | 2.4 |
| Secondary education (%), high | cold | -1.89 | 1.67 | -5.17 | 1.38 |
| Poverty (%), medium | cold | 5.32 | 2.15 | 1.11 | 9.54 |
| Poverty (%), high | cold | 6.38 | 2.66 | 1.18 | 11.59 |
| Gini, medium inequality | cold | 4.16 | 2.45 | -0.64 | 8.96 |
| Gini, high inequality | cold | 3.27 | 2.99 | -2.58 | 9.13 |
| Segregation, medium | cold | 3.52 | 1.82 | -0.04 | 7.08 |
| Segregation, high | cold | 5.75 | 2.3 | 1.24 | 10.25 |
| Population, medium | extreme cold | 0.12 | 0.13 | -0.14 | 0.38 |
| Population, high | extreme cold | 0.02 | 0.13 | -0.23 | 0.26 |
| Population density, medium | extreme cold | -0.06 | 0.13 | -0.31 | 0.19 |
| Population density, high | extreme cold | 0.07 | 0.16 | -0.25 | 0.38 |
| Built-up area (%), medium | extreme cold | 0.11 | 0.13 | -0.14 | 0.36 |
| Built-up area (%), high | extreme cold | 0.12 | 0.13 | -0.13 | 0.38 |
| Age-standardized mortality rate, medium | extreme cold | -0.44 | 0.13 | -0.7 | -0.17 |
| Age-standardized mortality rate, high | extreme cold | -0.18 | 0.15 | -0.47 | 0.11 |
| Living conditions score, medium | extreme cold | 0.02 | 0.14 | -0.26 | 0.3 |
| Living conditions score, low | extreme cold | 0.26 | 0.23 | -0.19 | 0.71 |
| Secondary education (%), medium | extreme cold | 0.02 | 0.14 | -0.25 | 0.29 |
| Secondary education (%), high | extreme cold | -0.17 | 0.14 | -0.45 | 0.11 |
| Poverty (%), medium | extreme cold | 0.1 | 0.19 | -0.27 | 0.47 |
| Poverty (%), high | extreme cold | 0.26 | 0.23 | -0.2 | 0.72 |
| Gini, medium inequality | extreme cold | 0.59 | 0.22 | 0.15 | 1.02 |
| Gini, high inequality | extreme cold | 0.41 | 0.26 | -0.1 | 0.93 |
| Segregation, medium | extreme cold | 0.15 | 0.17 | -0.18 | 0.49 |
| Segregation, high | extreme cold | -0.01 | 0.21 | -0.42 | 0.41 |
| Population, medium | extreme heat | -0.18 | 0.11 | -0.39 | 0.03 |
| Population, high | extreme heat | -0.17 | 0.1 | -0.37 | 0.03 |
| Population density, medium | extreme heat | 0.17 | 0.11 | -0.04 | 0.38 |
| Population density, high | extreme heat | -0.16 | 0.13 | -0.41 | 0.09 |
| Built-up area (%), medium | extreme heat | 0.08 | 0.11 | -0.12 | 0.29 |
| Built-up area (%), high | extreme heat | 0.00 | 0.1 | -0.2 | 0.21 |
| Age-standardized mortality rate, medium | extreme heat | 0.07 | 0.11 | -0.15 | 0.28 |
| Age-standardized mortality rate, high | extreme heat | 0.19 | 0.12 | -0.05 | 0.44 |
| Living conditions score, medium | extreme heat | -0.14 | 0.12 | -0.37 | 0.09 |
| Living conditions score, low | extreme heat | -0.01 | 0.19 | -0.38 | 0.35 |
| Secondary education (%), medium | extreme heat | -0.13 | 0.12 | -0.36 | 0.11 |
| Secondary education (%), high | extreme heat | -0.16 | 0.12 | -0.41 | 0.08 |
| Poverty (%), medium | extreme heat | -0.1 | 0.17 | -0.44 | 0.23 |
| Poverty (%), high | extreme heat | 0.06 | 0.21 | -0.35 | 0.47 |
| Gini, medium inequality | extreme heat | 0.12 | 0.16 | -0.19 | 0.43 |
| Gini, high inequality | extreme heat | -0.23 | 0.2 | -0.63 | 0.16 |
| Segregation, medium | extreme heat | 0.27 | 0.15 | -0.02 | 0.57 |
| Segregation, high | extreme heat | -0.05 | 0.19 | -0.43 | 0.33 |
| Population, medium | heat | -0.22 | 0.23 | -0.67 | 0.23 |
| Population, high | heat | -0.20 | 0.23 | -0.64 | 0.25 |
| Population density, medium | heat | 0.14 | 0.23 | -0.31 | 0.6 |
| Population density, high | heat | -0.6 | 0.3 | -1.18 | -0.01 |
| Built-up area (%), medium | heat | -0.19 | 0.23 | -0.65 | 0.26 |
| Built-up area (%), high | heat | -0.23 | 0.24 | -0.7 | 0.23 |
| Age-standardized mortality rate, medium | heat | 0.05 | 0.26 | -0.46 | 0.55 |
| Age-standardized mortality rate, high | heat | 0.00 | 0.29 | -0.56 | 0.56 |
| Living conditions score, medium | heat | -0.44 | 0.26 | -0.95 | 0.06 |
| Living conditions score, low | heat | -0.17 | 0.42 | -0.99 | 0.66 |
| Secondary education (%), medium | heat | 0.09 | 0.25 | -0.4 | 0.58 |
| Secondary education (%), high | heat | 0.05 | 0.27 | -0.48 | 0.57 |
| Poverty (%), medium | heat | -0.44 | 0.29 | -1.02 | 0.13 |
| Poverty (%), high | heat | -0.54 | 0.37 | -1.27 | 0.18 |
| Gini, medium inequality | heat | -0.20 | 0.36 | -0.91 | 0.51 |
| Gini, high inequality | heat | -1.07 | 0.45 | -1.95 | -0.2 |

Table S6. Interaction relative risks (IRRs) of all-cause mortality per 1°C more extreme cold and extreme hot temperatures among the population ages 65+ by levels of the socioeconomic and demographic characteristics of Latin American cities. The IRRs represent proportional difference in RR per 1°C associated with the given characteristic. RR for extreme cold was computed by dividing the difference in log-relative risk of mortality between temperatures at the 1^st^ and 5^th^ percentile of the city-specific daily mean temperature distribution by the difference in degrees Celsius between the 1^st^ percentile and 5^th^ percentile of the temperature distribution, and exponentiating the quotient. RR for heat was analogously obtained as the difference between the log-relative risk of mortality at the 99^th^ and 95^th^ percentile of the city-specific observed distribution of daily temperatures divided by the difference in degrees Celsius between the 99^th^ percentile and 95^th^ percentile of the temperature distribution, and exponentiating the quotient. For extreme cold, the IRR results can be interpreted as a change in the relative risk of mortality associated with a 1°C decrease in mean daily temperature below the 5^th^ percentile of the temperature distribution. For extreme heat, the IRR results present an estimated change in the relative risk of mortality associated with a 1°C increase in daily mean temperature above the 95^th^ percentile of the temperature distribution. Point estimates and 95% confidence intervals are obtained from the random effects meta-regressions that include a socioeconomic indicator, mean daily temperature, mean annual temperature range, climate zone, and country group. Separate meta-regressions were fitted for each socioeconomic indicator. The reference category for each effect modifier are cities with desirable levels of the indicator (e.g., low poverty, high living conditions score, etc.). In the case of population, population density, and % built-up area, the reference are cities with low absolute values (bottom tertile) of these characteristics. The analysis is based on 325 cities for all variables except poverty (n=319 cities), Gini index (n=296), and isolation index (n=303).

| Variable | IRR type | IRR Estimate | 95% CI Lower Bound | 95% CI Upper Bound |
| --- | --- | --- | --- | --- |
| Population, medium | extreme cold | 1.014 | 0.997 | 1.031 |
| Population, high | extreme cold | 1.005 | 0.990 | 1.020 |
| Population density, medium | extreme cold | 1.002 | 0.990 | 1.015 |
| Population density, high | extreme cold | 1.003 | 0.986 | 1.021 |
| Built-up area (%), medium | extreme cold | 0.999 | 0.984 | 1.015 |
| Built-up area (%), high | extreme cold | 1.002 | 0.988 | 1.016 |
| Age-standardized mortality rate, medium | extreme cold | 0.992 | 0.977 | 1.007 |
| Age-standardized mortality rate, high | extreme cold | 1.004 | 0.988 | 1.021 |
| Living conditions score, medium | extreme cold | 1.003 | 0.987 | 1.020 |
| Living conditions score, low | extreme cold | 0.990 | 0.966 | 1.015 |
| Secondary education (%), medium | extreme cold | 1.000 | 0.985 | 1.016 |
| Secondary education (%), high | extreme cold | 0.993 | 0.976 | 1.010 |
| Poverty (%), medium | extreme cold | 0.999 | 0.983 | 1.015 |
| Poverty (%), high | extreme cold | 0.982 | 0.961 | 1.005 |
| Gini index, medium inequality | extreme cold | 1.010 | 0.993 | 1.028 |
| Gini index, high inequality | extreme cold | 1.017 | 0.996 | 1.039 |
| Segregation, medium | extreme cold | 1.012 | 0.994 | 1.031 |
| Segregation, high | extreme cold | 1.016 | 0.992 | 1.041 |
| Population, medium | extreme heat | 0.993 | 0.965 | 1.021 |
| Population, high | extreme heat | 0.998 | 0.973 | 1.023 |
| Population density, medium | extreme heat | 1.014 | 0.993 | 1.035 |
| Population density, high | extreme heat | 0.994 | 0.967 | 1.022 |
| Built-up area (%), medium | extreme heat | 0.995 | 0.971 | 1.020 |
| Built-up area (%), high | extreme heat | 1.010 | 0.988 | 1.033 |
| Age-standardized mortality rate, medium | extreme heat | 0.992 | 0.977 | 1.007 |
| Age-standardized mortality rate, high | extreme heat | 1.004 | 0.988 | 1.021 |
| Living conditions score, medium | extreme heat | 1.001 | 0.974 | 1.029 |
| Living conditions score, low | extreme heat | 0.985 | 0.944 | 1.027 |
| Secondary education (%), medium | extreme heat | 0.995 | 0.970 | 1.020 |
| Secondary education (%), high | extreme heat | 0.985 | 0.958 | 1.012 |
| Poverty (%), medium | extreme heat | 1.000 | 0.976 | 1.024 |
| Poverty (%), high | extreme heat | 0.980 | 0.945 | 1.016 |
| Gini index, medium inequality | extreme heat | 1.020 | 0.990 | 1.052 |
| Gini index, high inequality | extreme heat | 1.011 | 0.975 | 1.049 |
| Segregation, medium | extreme heat | 1.001 | 0.968 | 1.035 |
| Segregation, high | extreme heat | 0.990 | 0.950 | 1.032 |

Table S7. Differences in excess death fractions (EDF) due to cold and hot temperatures for all ages for cardiovascular deaths by levels of the socioeconomic characteristics of Latin American cities. Refer to Table S2 for technical details.

| Variable | EDF type | | Estimate | | Std. Error | | 95% CI Lower Bound | | 95% CI Upper Bound | |  |
| --- | --- | --- | --- | --- | --- | --- | --- | --- | --- | --- | --- |
| Population, medium | | cold | | -0.125 | | 1.975 | | -3.996 | | 3.745 | |
| Population, high | | cold | | -5.340 | | 1.987 | | -9.234 | | -1.445 | |
| Population density, medium | | cold | | -1.043 | | 2.072 | | -5.104 | | 3.017 | |
| Population density, high | | cold | | 2.185 | | 2.647 | | -3.002 | | 7.373 | |
| Built-up area (%), medium | | cold | | -0.928 | | 2.029 | | -4.905 | | 3.048 | |
| Built-up area (%), high | | cold | | -2.718 | | 2.118 | | -6.869 | | 1.434 | |
| Age-standardized mortality rate, medium | | cold | | -4.651 | | 2.316 | | -9.191 | | -0.111 | |
| Age-standardized mortality rate, high | | cold | | 2.259 | | 3.081 | | -3.780 | | 8.299 | |
| Living conditions score, medium | | cold | | 5.330 | | 2.310 | | 0.802 | | 9.858 | |
| Living conditions score, low | | cold | | 6.128 | | 3.650 | | -1.026 | | 13.281 | |
| Secondary education (%), medium | | cold | | 0.947 | | 2.092 | | -3.153 | | 5.047 | |
| Secondary education (%), high | | cold | | -1.164 | | 2.301 | | -5.674 | | 3.346 | |
| Poverty (%), medium | | cold | | -0.822 | | 6.083 | | -12.744 | | 11.100 | |
| Poverty (%), high | | cold | | 0.433 | | 7.424 | | -14.117 | | 14.984 | |
| Gini, medium inequality | | cold | | 2.740 | | 2.754 | | -2.658 | | 8.138 | |
| Gini, high inequality | | cold | | 0.686 | | 3.350 | | -5.881 | | 7.252 | |
| Segregation, medium | | cold | | -2.204 | | 2.675 | | -7.446 | | 3.039 | |
| Segregation, high | | cold | | 0.064 | | 3.323 | | -6.450 | | 6.577 | |
| Population, medium | | heat | | -1.228 | | 0.875 | | -2.942 | | 0.487 | |
| Population, high | | heat | | -1.330 | | 0.887 | | -3.069 | | 0.409 | |
| Population density, medium | | heat | | 1.302 | | 0.914 | | -0.489 | | 3.092 | |
| Population density, high | | heat | | 0.340 | | 1.168 | | -1.950 | | 2.630 | |
| Built-up area (%), medium | | heat | | -0.879 | | 0.893 | | -2.630 | | 0.872 | |
| Built-up area (%), high | | heat | | -0.406 | | 0.938 | | -2.245 | | 1.433 | |
| Age-standardized mortality rate, medium | | heat | | 0.577 | | 1.038 | | -1.459 | | 2.612 | |
| Age-standardized mortality rate, high | | heat | | 2.129 | | 1.376 | | -0.569 | | 4.826 | |
| Living conditions score, medium | | heat | | -1.524 | | 1.013 | | -3.510 | | 0.462 | |
| Living conditions score, low | | heat | | 1.516 | | 1.608 | | -1.636 | | 4.669 | |
| Secondary education (%), medium | | heat | | -0.479 | | 0.925 | | -2.293 | | 1.334 | |
| Secondary education (%), high | | heat | | -1.166 | | 1.019 | | -3.163 | | 0.831 | |
| Poverty (%), medium | | heat | | -2.283 | | 1.174 | | -4.585 | | 0.018 | |
| Poverty (%), high | | heat | | -1.066 | | 1.436 | | -3.881 | | 1.750 | |
| Gini, medium inequality | | heat | | -1.580 | | 1.142 | | -3.819 | | 0.659 | |
| Gini, high inequality | | heat | | -2.078 | | 1.398 | | -4.819 | | 0.662 | |
| Segregation, medium | | heat | | 1.938 | | 1.016 | | -0.053 | | 3.929 | |
| Segregation, high | | heat | | 1.219 | | 1.265 | | -1.261 | | 3.698 | |


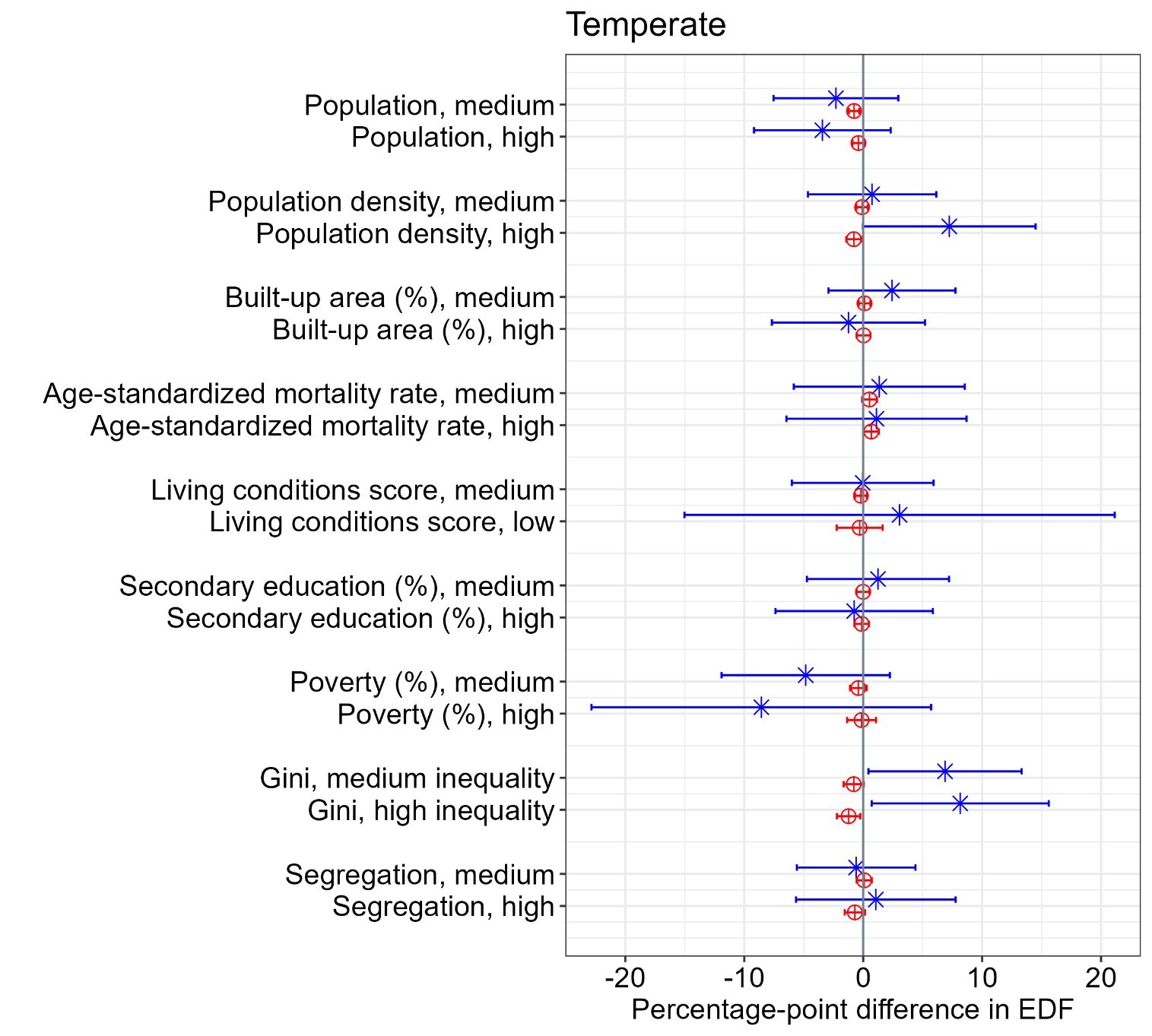


Figure S6. Differences in excess death fractions (EDF) associated with cold (in blue) and hot (in red) temperatures by levels of the socioeconomic and demographic characteristics among the cities in the temperate climate zone (n=112 cities).


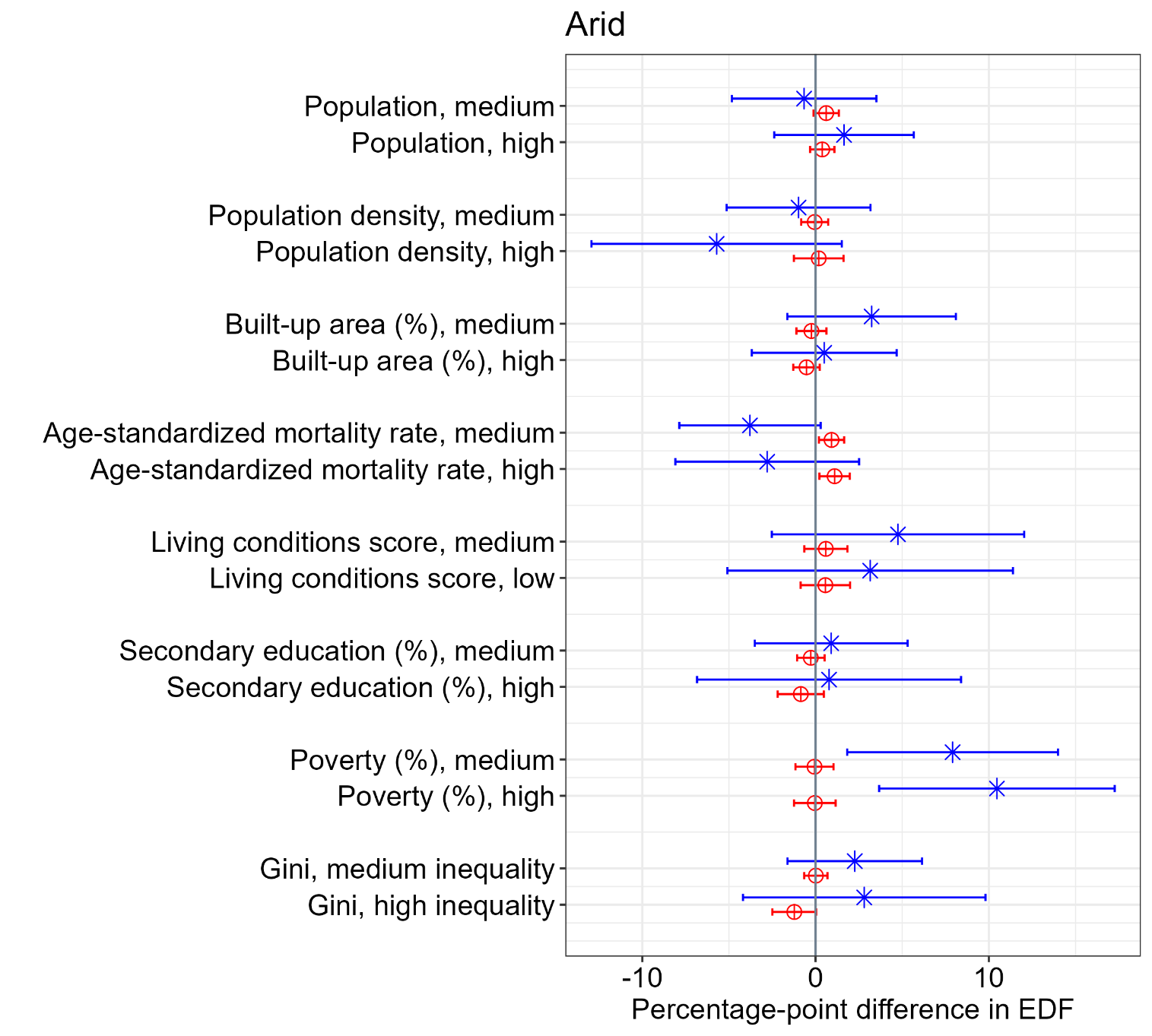


Figure S7. Differences in excess death fractions (EDF) associated with cold (in blue) and hot (in red) temperatures by levels of the socioeconomic and demographic characteristics among the cities in the arid climate zone (n=79 cities). There was not enough variability in the segregation measure to model it as a categorical effect modifier for the arid cities.


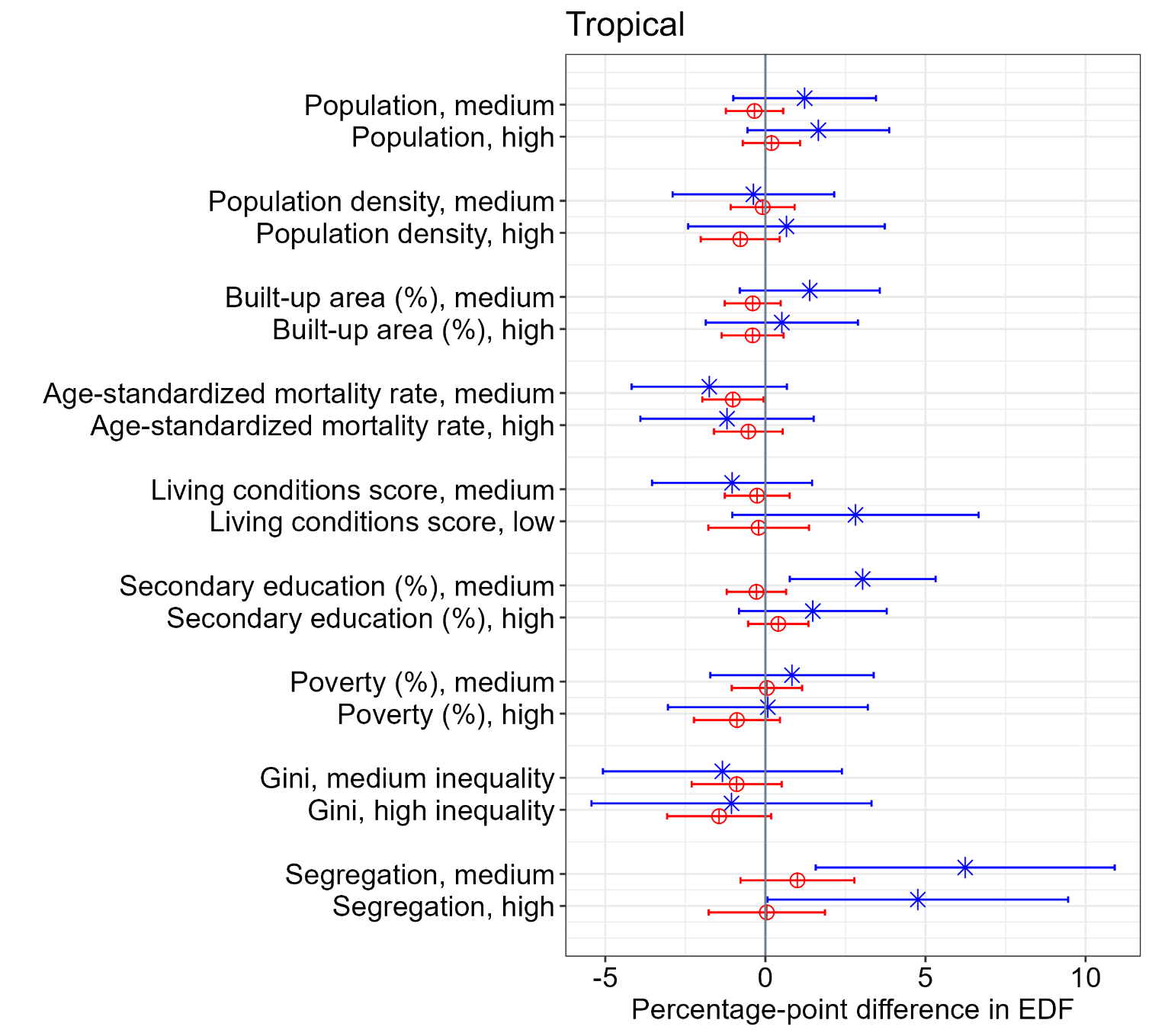


Figure S8. Differences in excess death fractions (EDF) associated with cold (in blue) and hot (in red) temperatures by levels of the socioeconomic and demographic characteristics among the cities in the tropical climate zone (n=134 cities).


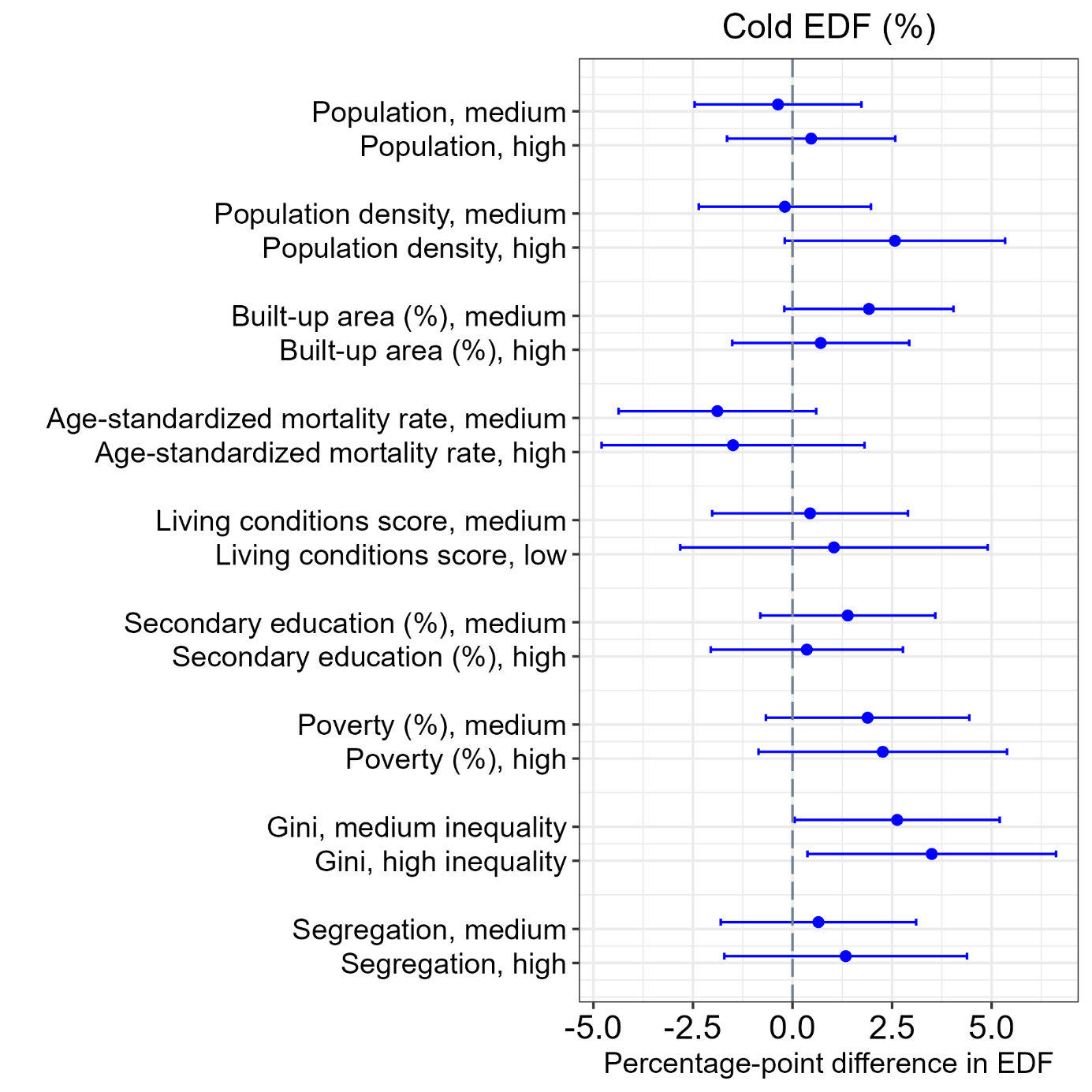

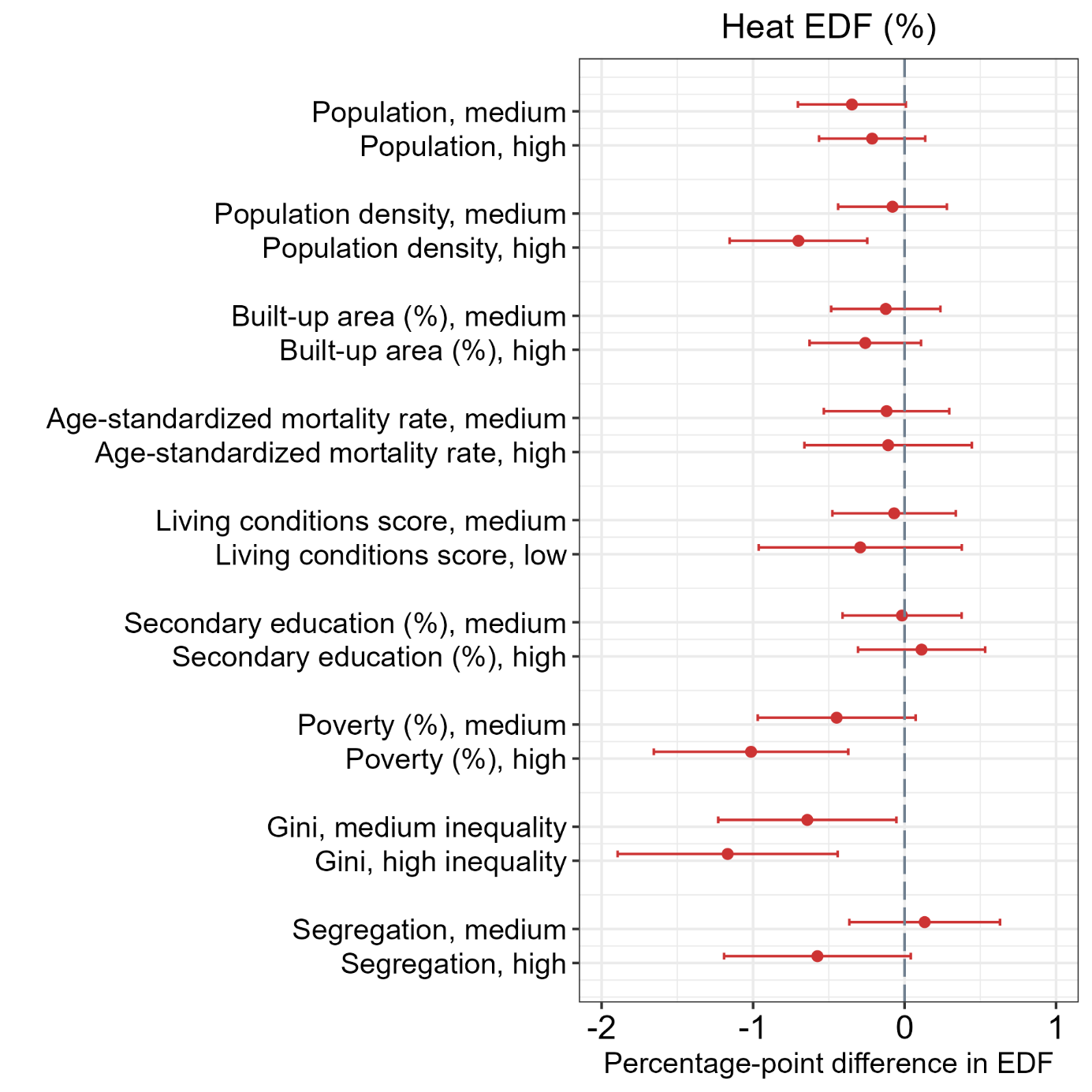


# Figure S9. Differences in excess death fractions (EDF) of all-cause mortality associated with cold and hot temperatures by levels of the socioeconomic and demographic characteristics of Latin American cities, adjusted by the proportion of city population 65 years and older. Refer to Table S2 for technical details.

# Figure S10. Differences in excess death fractions (EDF) of all-cause mortality associated with extremely cold and extremely hot temperatures by levels of the socioeconomic and demographic characteristics of Latin American cities, adjusted by the proportion of city population 65 years and older. Refer to Table S2 for technical details.


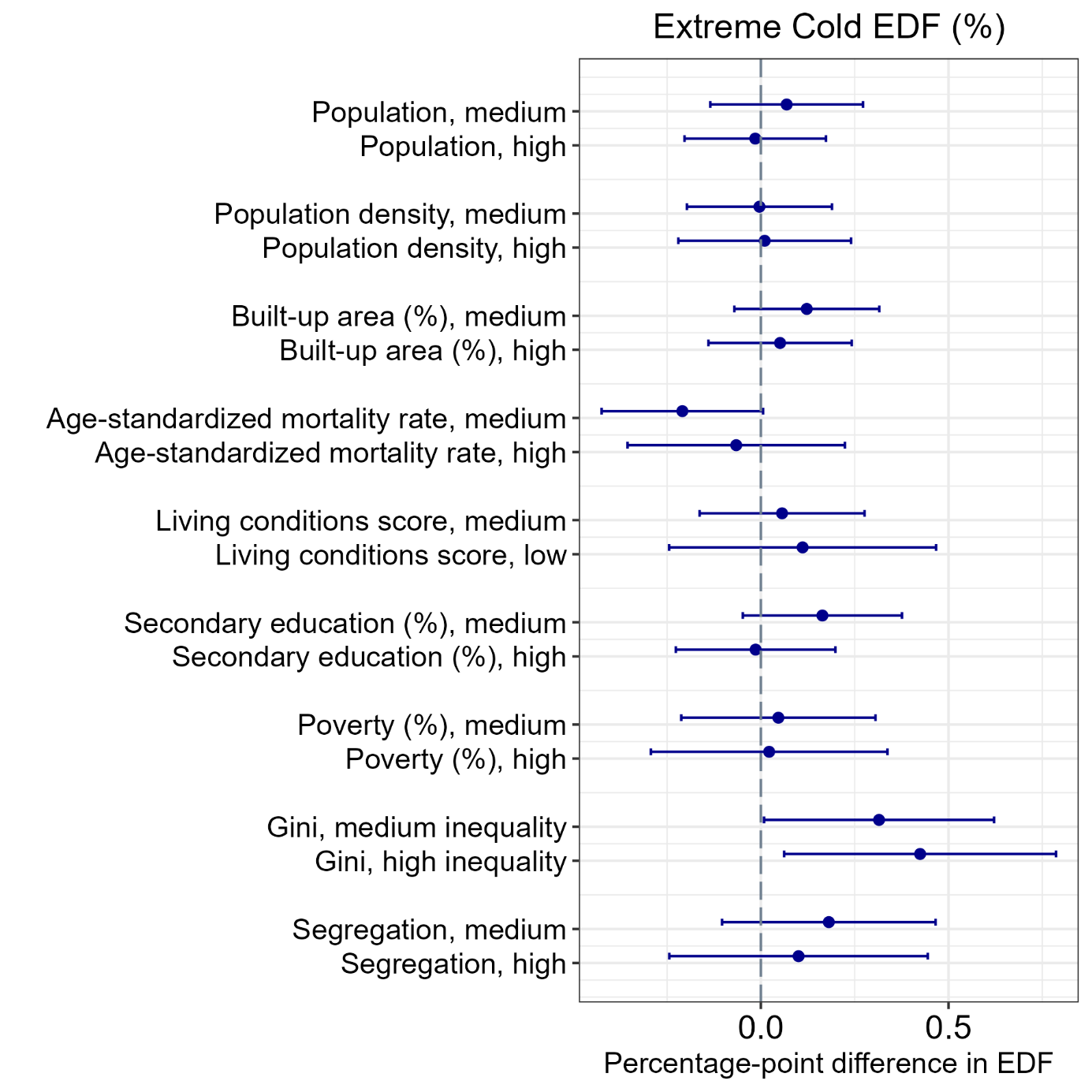

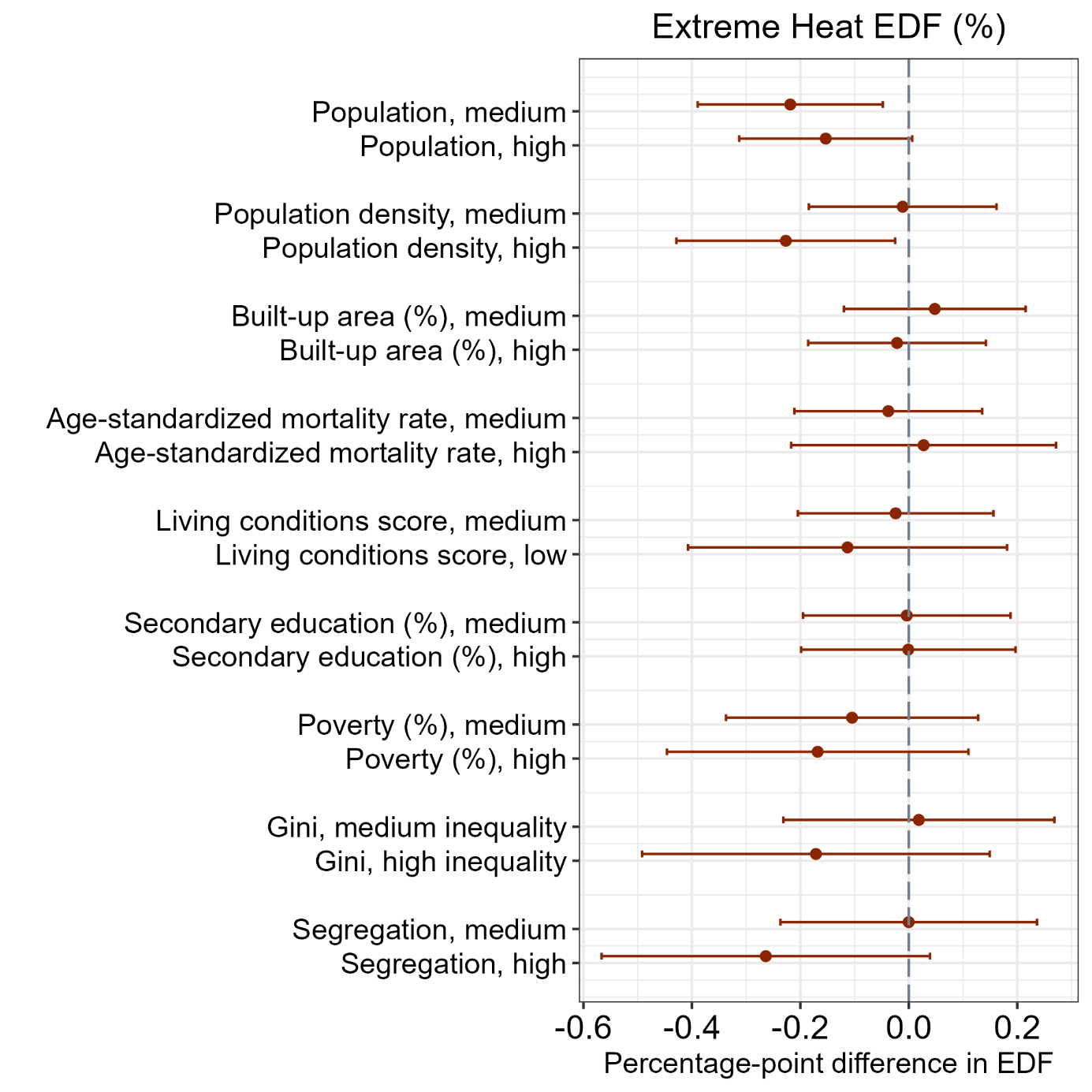


Table S8. Differences in excess death fractions (EDF) due to cold, hot, extremely cold, and extremely hot temperatures among all ages by levels of socioeconomic and demographic characteristics of Latin American cities, adjusted by the proportion of city population 65 years and older. Refer to Table S2 for technical details.

| Variable | EDF type | Estimate | Std. Error | 95% CI Lower Bound | 95% CI Upper Bound |
| --- | --- | --- | --- | --- | --- |
| Population, medium | cold | -0.363 | 1.069 | -2.458 | 1.731 |
| Population, high | cold | 0.470 | 1.079 | -1.644 | 2.584 |
| Population density, medium | cold | -0.191 | 1.103 | -2.353 | 1.972 |
| Population density, high | cold | 2.574 | 1.411 | -0.192 | 5.340 |
| Built-up area (%), medium | cold | 1.920 | 1.084 | -0.205 | 4.045 |
| Built-up area (%), high | cold | 0.709 | 1.134 | -1.514 | 2.931 |
| Age-standardized mortality rate, medium | cold | -1.886 | 1.266 | -4.366 | 0.595 |
| Age-standardized mortality rate, high | cold | -1.492 | 1.685 | -4.794 | 1.810 |
| Living conditions score, medium | cold | 0.442 | 1.255 | -2.017 | 2.901 |
| Living conditions score, low | cold | 1.042 | 1.970 | -2.820 | 4.903 |
| Secondary education (%), medium | cold | 1.389 | 1.121 | -0.808 | 3.585 |
| Secondary education (%), high | cold | 0.361 | 1.231 | -2.052 | 2.773 |
| Poverty (%), medium | cold | 1.888 | 1.304 | -0.667 | 4.443 |
| Poverty (%), high | cold | 2.268 | 1.591 | -0.851 | 5.387 |
| Gini, medium inequality | cold | 2.630 | 1.314 | 0.054 | 5.205 |
| Gini, high inequality | cold | 3.498 | 1.593 | 0.375 | 6.620 |
| Segregation, medium | cold | 0.651 | 1.252 | -1.802 | 3.104 |
| Segregation, high | cold | 1.338 | 1.555 | -1.710 | 4.385 |
| Population, medium | extreme cold | 0.069 | 0.104 | -0.135 | 0.272 |
| Population, high | extreme cold | -0.015 | 0.096 | -0.204 | 0.174 |
| Population density, medium | extreme cold | -0.004 | 0.099 | -0.197 | 0.189 |
| Population density, high | extreme cold | 0.010 | 0.117 | -0.220 | 0.240 |
| Built-up area (%), medium | extreme cold | 0.122 | 0.099 | -0.071 | 0.315 |
| Built-up area (%), high | extreme cold | 0.051 | 0.097 | -0.140 | 0.242 |
| Age-standardized mortality rate, medium | extreme cold | -0.209 | 0.110 | -0.425 | 0.006 |
| Age-standardized mortality rate, high | extreme cold | -0.066 | 0.148 | -0.355 | 0.224 |
| Living conditions score, medium | extreme cold | 0.056 | 0.112 | -0.163 | 0.276 |
| Living conditions score, low | extreme cold | 0.111 | 0.182 | -0.245 | 0.467 |
| Secondary education (%), medium | extreme cold | 0.164 | 0.108 | -0.048 | 0.376 |
| Secondary education (%), high | extreme cold | -0.014 | 0.108 | -0.227 | 0.199 |
| Poverty (%), medium | extreme cold | 0.047 | 0.132 | -0.212 | 0.306 |
| Poverty (%), high | extreme cold | 0.022 | 0.161 | -0.293 | 0.337 |
| Gini, medium inequality | extreme cold | 0.315 | 0.156 | 0.008 | 0.621 |
| Gini, high inequality | extreme cold | 0.424 | 0.185 | 0.062 | 0.787 |
| Segregation, medium | extreme cold | 0.181 | 0.145 | -0.103 | 0.465 |
| Segregation, high | extreme cold | 0.100 | 0.176 | -0.244 | 0.445 |
| Population, medium | extreme heat | -0.219 | 0.087 | -0.389 | -0.048 |
| Population, high | extreme heat | -0.153 | 0.081 | -0.313 | 0.006 |
| Population density, medium | extreme heat | -0.011 | 0.088 | -0.184 | 0.162 |
| Population density, high | extreme heat | -0.227 | 0.103 | -0.429 | -0.025 |
| Built-up area (%), medium | extreme heat | 0.048 | 0.086 | -0.120 | 0.216 |
| Built-up area (%), high | extreme heat | -0.022 | 0.084 | -0.186 | 0.142 |
| Age-standardized mortality rate, medium | extreme heat | -0.038 | 0.088 | -0.211 | 0.135 |
| Age-standardized mortality rate, high | extreme heat | 0.027 | 0.125 | -0.217 | 0.272 |
| Living conditions score, medium | extreme heat | -0.024 | 0.092 | -0.205 | 0.156 |
| Living conditions score, low | extreme heat | -0.113 | 0.150 | -0.407 | 0.181 |
| Secondary education (%), medium | extreme heat | -0.004 | 0.098 | -0.195 | 0.188 |
| Secondary education (%), high | extreme heat | -0.001 | 0.101 | -0.199 | 0.197 |
| Poverty (%), medium | extreme heat | -0.105 | 0.119 | -0.337 | 0.128 |
| Poverty (%), high | extreme heat | -0.168 | 0.142 | -0.446 | 0.110 |
| Gini, medium inequality | extreme heat | 0.018 | 0.128 | -0.231 | 0.268 |
| Gini, high inequality | extreme heat | -0.171 | 0.164 | -0.492 | 0.149 |
| Segregation, medium | extreme heat | 0.000 | 0.121 | -0.237 | 0.236 |
| Segregation, high | extreme heat | -0.264 | 0.155 | -0.567 | 0.039 |
| Population, medium | heat | -0.347 | 0.182 | -0.704 | 0.009 |
| Population, high | heat | -0.214 | 0.179 | -0.565 | 0.136 |
| Population density, medium | heat | -0.080 | 0.183 | -0.439 | 0.279 |
| Population density, high | heat | -0.701 | 0.232 | -1.155 | -0.246 |
| Built-up area (%), medium | heat | -0.124 | 0.184 | -0.485 | 0.237 |
| Built-up area (%), high | heat | -0.259 | 0.188 | -0.627 | 0.109 |
| Age-standardized mortality rate, medium | heat | -0.119 | 0.211 | -0.533 | 0.295 |
| Age-standardized mortality rate, high | heat | -0.108 | 0.282 | -0.661 | 0.444 |
| Living conditions score, medium | heat | -0.069 | 0.208 | -0.476 | 0.338 |
| Living conditions score, low | heat | -0.292 | 0.342 | -0.962 | 0.378 |
| Secondary education (%), medium | heat | -0.017 | 0.201 | -0.410 | 0.376 |
| Secondary education (%), high | heat | 0.112 | 0.214 | -0.307 | 0.531 |
| Poverty (%), medium | heat | -0.448 | 0.266 | -0.969 | 0.073 |
| Poverty (%), high | heat | -1.013 | 0.328 | -1.655 | -0.371 |
| Gini, medium inequality | heat | -0.642 | 0.300 | -1.230 | -0.054 |
| Gini, high inequality | heat | -1.168 | 0.370 | -1.894 | -0.442 |


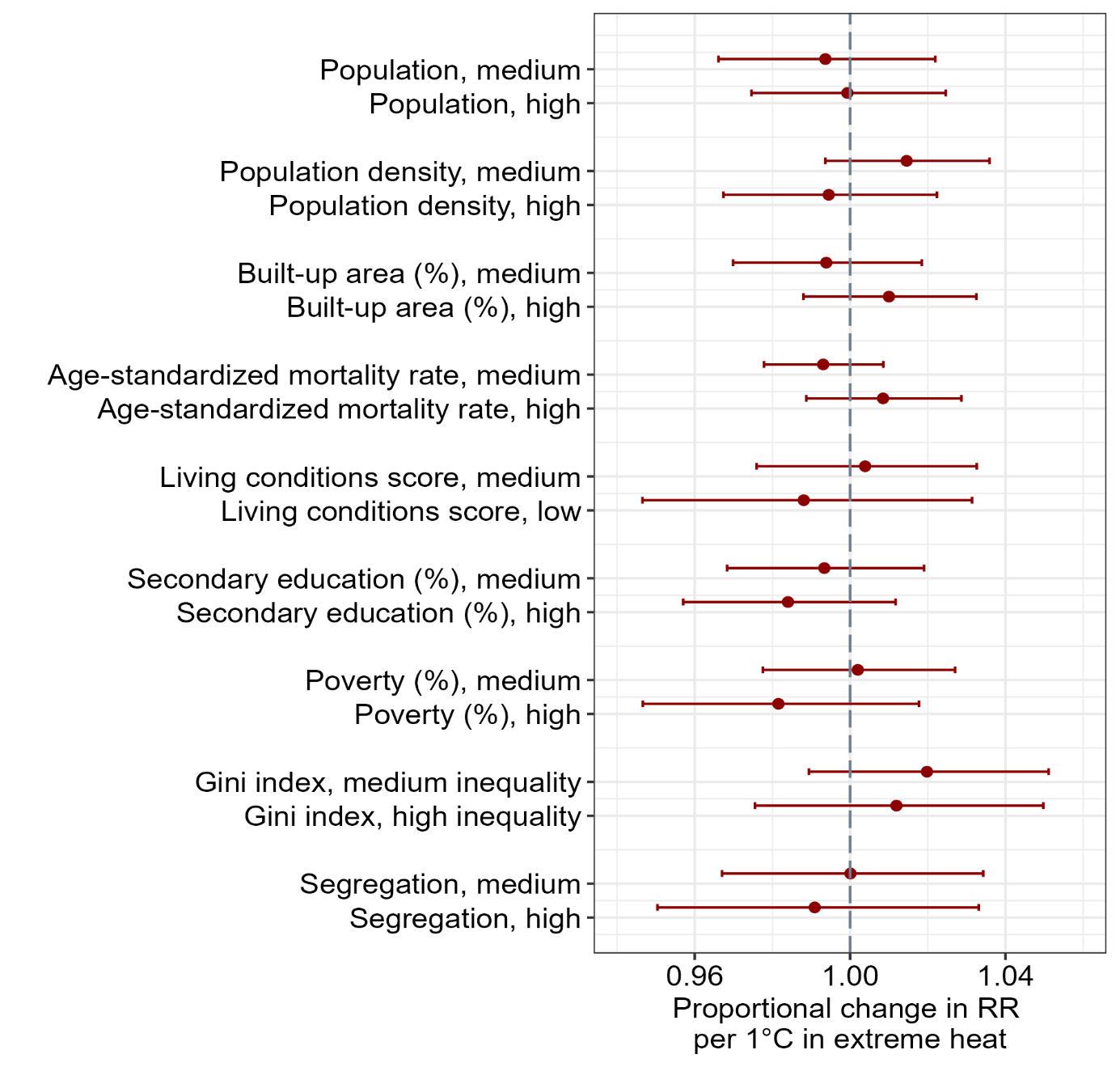

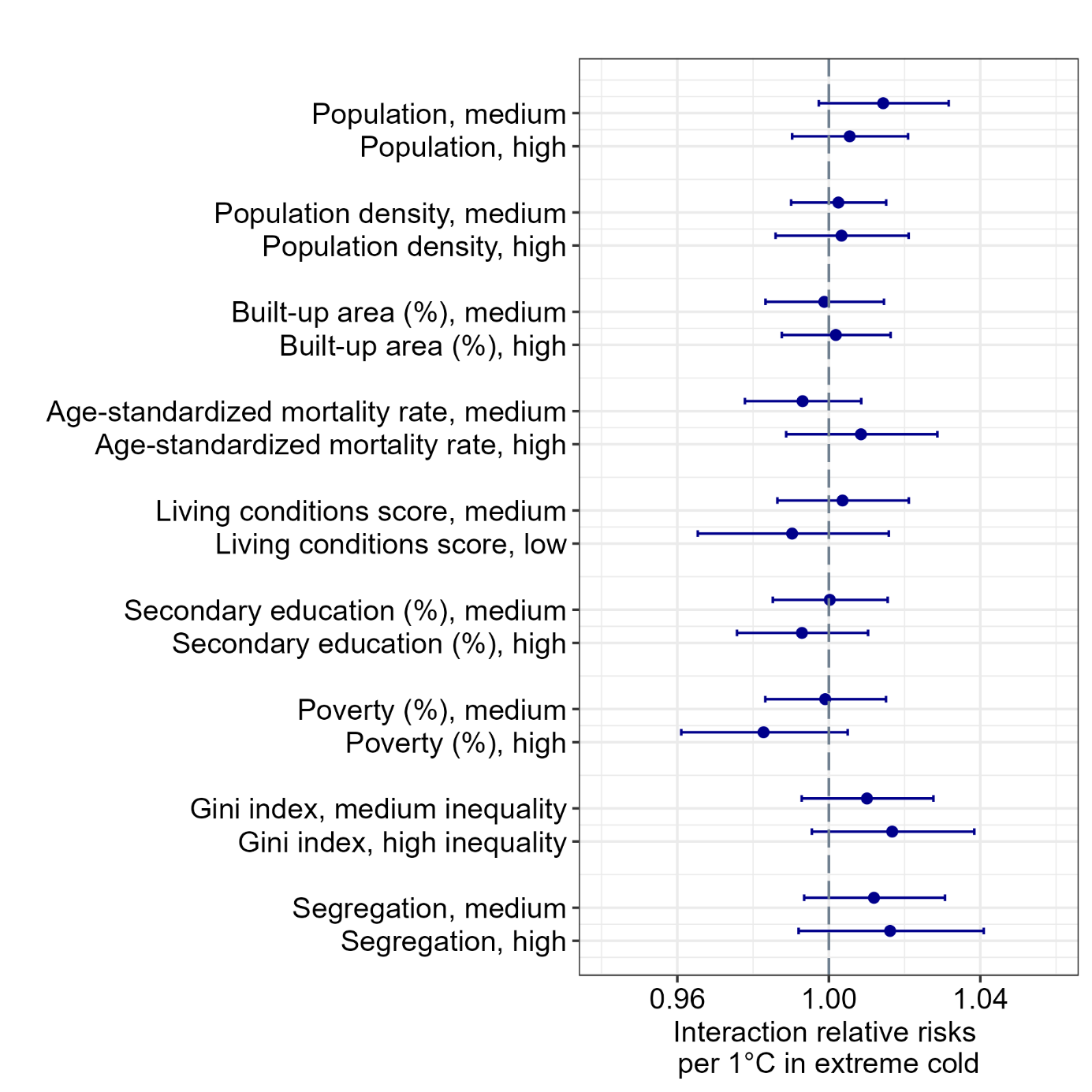


# Figure S11. Interaction relative risks (IRRs) of all-cause mortality per 1°C more extreme cold and extreme hot temperatures by levels of the socioeconomic characteristics of Latin American cities, adjusted by the proportion of city population 65 years and older. Refer to Table S9 for technical details.

Table S9. Interaction relative risks (IRRs) of all-cause mortality per 1°C more extreme cold and extreme hot temperatures by levels of the socioeconomic characteristics of Latin American cities, adjusted by the proportion of city population 65 years and older. The IRRs represent proportional difference in RR per 1°C associated with the given characteristic. RR for extreme cold was computed by dividing the difference in log-relative risk of mortality between temperatures at the 1^st^ and 5^th^ percentile of the city-specific daily mean temperature distribution by the difference in degrees Celsius between the 1^st^ percentile and 5^th^ percentile of the temperature distribution, and exponentiating the quotient. RR for heat was analogously obtained as the difference between the log-relative risk of mortality at the 99^th^ and 95^th^ percentile of the city-specific observed distribution of daily temperatures divided by the difference in degrees Celsius between the 99^th^ percentile and 95^th^ percentile of the temperature distribution, and exponentiating the quotient. For extreme cold, the IRR results can be interpreted as a change in the relative risk of mortality associated with a 1°C decrease in mean daily temperature below the 5^th^ percentile of the temperature distribution. For extreme heat, the IRR results present an estimated change in the relative risk of mortality associated with a 1°C increase in daily mean temperature above the 95^th^ percentile of the temperature distribution. Point estimates and 95% confidence intervals are obtained from the random effects meta-regressions that include a socioeconomic indicator, mean daily temperature, mean annual temperature range, climate zone, country group, and proportion of city population older than 65 years. Separate meta-regressions were fitted for each socioeconomic indicator. The reference category for each socioeconomic effect modifier are cities with desirable levels of the indicator (e.g., low poverty, high living conditions score, etc.). In the case of population, population density, and % built-up area, the reference are cities with low absolute values (bottom tertile) of these characteristics. The analysis is based on 325 cities for all variables except poverty (n=319 cities), Gini index (n=296), and isolation index (n=303). Refer to Table 1 for variables’ definition.

| Variable | IRR type | IRR Estimate | Std. Error | 95% CI Lower Bound | 95% CI Upper Bound |
| --- | --- | --- | --- | --- | --- |
| Population, medium | extreme cold | 1.014 | 0.997 | 1.032 | 1.014 |
| Population, high | extreme cold | 1.006 | 0.990 | 1.021 | 1.006 |
| Population density, medium | extreme cold | 1.003 | 0.990 | 1.015 | 1.003 |
| Population density, high | extreme cold | 1.003 | 0.986 | 1.021 | 1.003 |
| Built-up area (%), medium | extreme cold | 0.999 | 0.983 | 1.015 | 0.999 |
| Built-up area (%), high | extreme cold | 1.002 | 0.988 | 1.016 | 1.002 |
| Age-standardized mortality rate, medium | extreme cold | 0.993 | 0.978 | 1.009 | 0.993 |
| Age-standardized mortality rate, high | extreme cold | 1.008 | 0.989 | 1.029 | 1.008 |
| Living conditions score, medium | extreme cold | 1.004 | 0.986 | 1.021 | 1.004 |
| Living conditions score, low | extreme cold | 0.990 | 0.965 | 1.016 | 0.990 |
| Secondary education (%), medium | extreme cold | 1.000 | 0.985 | 1.016 | 1.000 |
| Secondary education (%), high | extreme cold | 0.993 | 0.976 | 1.010 | 0.993 |
| Poverty (%), medium | extreme cold | 0.999 | 0.983 | 1.015 | 0.999 |
| Poverty (%), high | extreme cold | 0.983 | 0.961 | 1.005 | 0.983 |
| Gini index, medium inequality | extreme cold | 1.010 | 0.993 | 1.028 | 1.010 |
| Gini index, high inequality | extreme cold | 1.017 | 0.996 | 1.038 | 1.017 |
| Segregation, medium | extreme cold | 1.012 | 0.993 | 1.031 | 1.012 |
| Segregation, high | extreme cold | 1.016 | 0.992 | 1.041 | 1.016 |
| Population, medium | extreme heat | 0.994 | 0.966 | 1.022 | 0.994 |
| Population, high | extreme heat | 0.999 | 0.975 | 1.025 | 0.999 |
| Population density, medium | extreme heat | 1.015 | 0.994 | 1.036 | 1.015 |
| Population density, high | extreme heat | 0.995 | 0.967 | 1.022 | 0.995 |
| Built-up area (%), medium | extreme heat | 0.994 | 0.970 | 1.018 | 0.994 |
| Built-up area (%), high | extreme heat | 1.010 | 0.988 | 1.033 | 1.010 |
| Age-standardized mortality rate, medium | extreme heat | 0.993 | 0.978 | 1.009 | 0.993 |
| Age-standardized mortality rate, high | extreme heat | 1.008 | 0.989 | 1.029 | 1.008 |
| Living conditions score, medium | extreme heat | 1.004 | 0.976 | 1.033 | 1.004 |
| Living conditions score, low | extreme heat | 0.988 | 0.947 | 1.031 | 0.988 |
| Secondary education (%), medium | extreme heat | 0.993 | 0.968 | 1.019 | 0.993 |
| Secondary education (%), high | extreme heat | 0.984 | 0.957 | 1.012 | 0.984 |
| Poverty (%), medium | extreme heat | 1.002 | 0.978 | 1.027 | 1.002 |
| Poverty (%), high | extreme heat | 0.982 | 0.947 | 1.018 | 0.982 |
| Gini index, medium inequality | extreme heat | 1.020 | 0.989 | 1.051 | 1.020 |
| Gini index, high inequality | extreme heat | 1.012 | 0.976 | 1.050 | 1.012 |

Table S10. Associations between minimum mortality temperature (MMT) and city-level characteristics in Latin American cities.

| Variable | Estimate | Std. Error | p-value |
| --- | --- | --- | --- |
| Population, medium | -0.022 | 0.146 | 0.882 |
| Population, high | 0.051 | 0.148 | 0.731 |
| Population density, medium | -0.242 | 0.150 | 0.108 |
| Population density, high | 0.311 | 0.192 | 0.106 |
| **Built-up area (%), medium** | **-0.360** | **0.148** | **0.015** |
| Built-up area (%), high | -0.215 | 0.154 | 0.164 |
| Age-standardized mortality rate, medium | 0.151 | 0.163 | 0.353 |
| Age-standardized mortality rate, high | -0.014 | 0.179 | 0.938 |
| Living conditions score, medium | -0.019 | 0.165 | 0.909 |
| Living conditions score, low | -0.317 | 0.258 | 0.221 |
| Secondary education (%), medium | 0.130 | 0.153 | 0.397 |
| Secondary education (%), high | -0.087 | 0.168 | 0.603 |
| Poverty (%), medium | 0.231 | 0.167 | 0.169 |
| Poverty (%), high | 0.087 | 0.207 | 0.674 |
| **Gini index, medium inequality** | **0.510** | **0.204** | **0.013** |
| **Gini index, high inequality** | **0.705** | **0.251** | **0.005** |
| Segregation, medium | -0.026 | 0.207 | 0.900 |
| Segregation, high | 0.364 | 0.263 | 0.166 |

Statistically significant associations (p<0.05) are **bolded**

Separate OLS regressions were used to obtain associations between MMT and each city-level variable (modeled as categorical variables). The reference category for each variable are cities with desirable levels of the indicator (e.g., low poverty, high living conditions, etc.). In the case of population, population density, and % urban area, the reference are cities with low (bottom tertile) values of these characteristics. Refer to Table 1 in the main text for variables’ definition. The analysis is based on 325 cities for all variables except poverty (n=319 cities), Gini index (n=296), and segregation (n=303). The models adjusted for country group, mean daily temperature, annual average temperature range, and climate zone.
